# β-Cyclodextrin Inclusion Complexes with Catechol-Containing Antioxidants Protocatechuic Aldehyde and Protocatechuic Acid—An Atomistic Perspective on Structural and Thermodynamic Stabilities

**DOI:** 10.3390/molecules26123574

**Published:** 2021-06-11

**Authors:** Thammarat Aree

**Affiliations:** Department of Chemistry, Faculty of Science, Chulalongkorn University, Bangkok 10330, Thailand; athammar@chula.ac.th; Tel.: +66-2-218-7584; Fax: +66-2-218-7598

**Keywords:** β-cyclodextrin, inclusion complex, protocatechuic aldehyde, protocatechuic acid, X-ray analysis, density functional theory, hydrogen bond, catechol, antioxidant

## Abstract

Protocatechuic aldehyde (PCAL) and protocatechuic acid (PCAC) are catechol derivatives and have broad therapeutic effects associated with their antiradical activity. Their pharmacological and physicochemical properties have been improved via the cyclodextrin (CD) encapsulation. Because the characteristics of β-CD inclusion complexes with PCAL (**1**) and PCAC (**2**) are still equivocal, we get to the bottom of the inclusion complexation by an integrated study of single-crystal X-ray diffraction and DFT full-geometry optimization. X-ray analysis unveiled that PCAL and PCAC are nearly totally shielded in the β-CD wall. Their aromatic rings are vertically aligned in the β-CD cavity such that the functional groups on the opposite side of the ring (3,4-di(OH) and 1-CHO/1-COOH groups) are placed nearby the O6–H and O2–H/O3–H rims, respectively. The preferred inclusion modes in **1** and **2** help to establish crystal contacts of OH⋅⋅⋅O H-bonds with the adjacent β-CD OH groups and water molecules. By contrast, the DFT-optimized structures of both complexes in the gas phase are thermodynamically stable via the four newly formed host–guest OH⋯O H-bonds. The intermolecular OH⋅⋅⋅O H-bonds between PCAL/PCAC 3,4-di(OH) and β-CD O6–H groups, and the shielding of OH groups in the β-CD wall help to stabilize these antioxidants in the β-CD cavity, as observed in our earlier studies. Moreover, PCAL and PCAC in distinct lattice environments are compared for insights into their structural flexibility.

## 1. Introduction

**Scheme 1:**
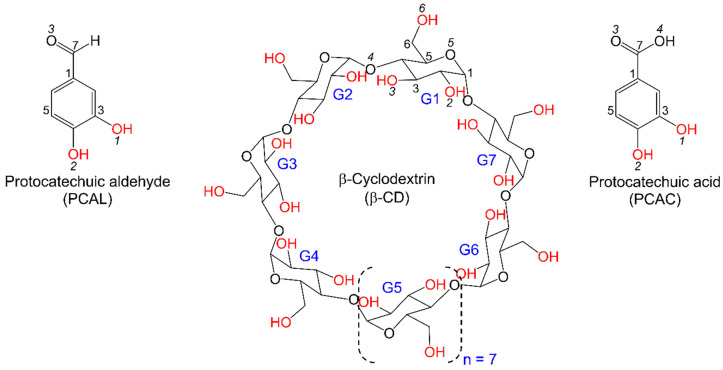
Chemical structures and atom labeling schemes of PCAL, PCAC, and β-CD molecules; OH groups are in red.

Protocatechuic acid (3,4-dihydroxybenzoic acid; PCAC, Scheme 1) is a phenolic acid present in various edible and medicinal plants, for example, green tea [1], acai fruit oil [2], roselle [3], and mushrooms [4]. PCAC is the primary human metabolite of anthocyanins, which are complex polyphenols [5]. Protocatechuic aldehyde (3,4-dihydroxybenzaldehyde; PCAL, Scheme 1) is a phenolic aldehyde found in the mushroom *Phellinus linteus* [6], the herb *S. miltiorrhiza* [7], and barley tea [8]. PCAC is a main metabolite of PCAL in rat plasma [9]. PCAC, PCAL, and other polyphenols like gallic, vanillic, caffeic, and ferulic acids are also found in cork [10]. Both catechol-containing compounds exhibit a wide range of pharmacological activities, for example, antioxidant, anti-inflammatory, and anticancer activities for PCAC [11,12,13,14,15,16] and for PCAL [17,18]. Moreover, PCAC and PCAL are most abundant in *Salvia miltiorrhiza*, a traditional Chinese herbal drug for the treatment of heart diseases [12,13,19,20]. Particular attention is paid for the free radical scavenging activity. Comparing the antioxidant capacity among different phenolic acids, the DPPH-derived percentage of antioxidant activity increases with increasing number of carrying free OH groups: PCAC, 41%, 2 OH’s ≈ caffeic acid (CFA), 44%, 2 OHs < gallic acid, 75%, 3 OHs < chlorogenic acid (CGA), 93%, 5 OHs [21]. The antioxidant activity in vitro of PCAC is ~3 times more potent than that of Trolox, based on the DPPH assay [22] because PCAC (as well as other o-diphenols) promptly converts to the corresponding stable o-quinones [23].

α-, β- and γ-Cyclodextrins (CDs) are macrocyclic carbohydrates consisting of 6, 7, and 8 D-glucose units, respectively [24,25], Scheme 1. They resemble a hollow, truncated cone and are powerful encapsulating agents for improvement of stabilities, activities, and solubilities of various bioactive compounds [26]. A number of studies have been carried out on the equimolar inclusion complexes of β-CD with PCAC and PCAL. Olive polyphenols including tyrosol, hydroxytyrosol (HTY), and PCAC form 1:1 inclusion complexes with β-CD with binding constants (*K*_a_’s) of 497, 355, and 661 M^−1^, respectively, based on NMR data [27]. A combined study of molecular dynamics and semiempirical PM3 calculation has predicted that the most stable inclusion structure of β-CD–PCAC has the phenyl ring included in the β-CD cavity and the COOH group protruded from the O2/O3 end; the opposite inclusion mode is less stable by 9.44 kcal mol^−1^ [27]. The relatively stable complex of β-CD with apple PCAC has *K*_a_ = 264 ± 29 M^−1^, as derived from a UV-binding study [28]. Based on NMR data, PCAL, CFA, ferulic acid and vanillin form 1:1 inclusion complexes with β-CD and have a common inclusion geometry with the aromatic ring embedded in the β-CD cavity and the CHO and COOH moieties protruded from the O2/O3 end [29]. Among the complexes with substituted phenols, the β-CD–PCAL complex has the highest *K*_a_ = 6700 ± 777 M^−1^, as deduced from UV-visible spectroscopy [29]. Moreover, through the encapsulation process, β-CD has been used as modifier in capillary zone electrophoresis to improve separation of seven phenolic acids (for example, PCAL, PCAC, rosmarinic acid, etc.) in three *Salvia* species [30]. The physicochemical properties of polyphenols (epicatechin, quercetin, CGA, PCAC, CFA, etc.) in plant extracts have been improved via CD inclusion complexation, for example, the thermal stability of H. sabdariffa L. anthocyanin extract by β-CD [31], the water solubility of propolis extract by β-CD [32], the thermal stability and antioxidant of St. John’s wort extract by β-CD [33], and the oxidative stability of apple pomace extract by hydroxypropyl-β-CD (HP-β-CD) [34].

The literature review above together with our recently reported crystal structures of β-CD complexes with catechol-containing antioxidants in olive [35] raise several questions, leading to a twofold hypothesis of this work. (i) Because PCAC and PCAL are highly structurally related and have an optimum size for the β-CD nanocavity, they probably form an equimolar complex with β-CD with a common inclusion of the aromatic moiety and the protrusion of 3,4-di(OH) group from the O6 side. (ii) Both complexes are thermodynamically stable due to the optimal host–guest interactions and the molecular stabilities of PCAC and PCAL are improved upon inclusion in the β-CD cavity. To validate the hypothesis statements and gain an atomic-level understanding on inclusion complexation, we investigate the β-CD encapsulation of PCAL (**1**) and PCAC (**2**) by a combined study of single-crystal X-ray diffraction and density functional theory (DFT) full-geometry optimization. Plus, PCAL and PCAC seem to be rather rigid. We further explore to what extent the two antioxidants adapt their rotatable CHO and COOH groups to distinct lattice circumstances (in the free form, encapsulated in β-CD, and in complex with protein).

## 2. Results and Discussion

**Figure 1:**
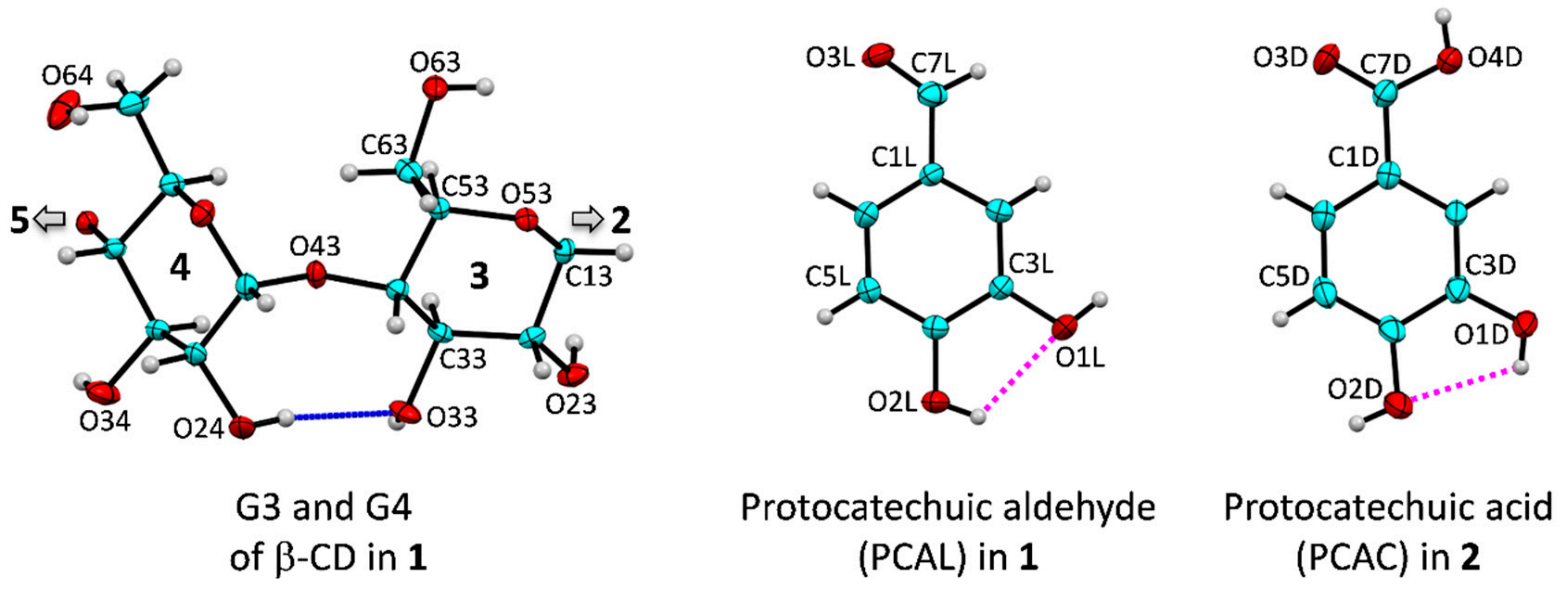
Atom numbering of β-CD, PCAL and PCAC molecules; ORTEP plot at 30% probability level. The intramolecular, interglucose O3(*n*)···O2(*n* + 1) hydrogen bonds stabilizing the annular conformation of β-CD macrocycle are shown for glucose units G3 and G4 (blue connecting line); for a complete molecule, see Figure 4. PCAL and PCAC are stabilized by intramolecular O2L–H···O1L and O1D–H···O2D hydrogen bonds, respectively (magenta dotted lines).

Here, we adopt the atom numbering scheme as used in our previous works [36,37,38,39]. β-CD is conventionally numbered as carbohydrates, viz., atoms C63–O63 denote the methylene C6−H_2_ connected to hydroxyl O6−H groups of glucose unit 3 (G3) in the complex β-CD−PCAL (**1**), Figure 1. PCAL and PCAC are numbered according to the corresponding IUPAC names and are additionally labeled with arbitrary letters L and D in the respective complexes **1** and **2**, Figure 1.

### 2.1. β-CD Macrocycles Are Affected to Some Extent by Inclusion of PCAL and PCAC

The molecular structures of CD hydrates are usually round. The uncomplexed α-, β-, and γ-CDs have respective pseudo-6-, 7- and 8-fold rotational axes passing through the molecular centroid and perpendicular to the molecular plane (O4 plane). This is due to the small differences of the composing glucose units, particularly the rotatable O6–H (freely) and O2–H/O3–H (restricted due to the interglucose H-bonds) groups lining the narrower and wider perimeters, respectively. From one to another inclusion complex, CD macrocycles adapt to some extent to optimally fit to various aromatic guest molecules. Therefore, the host–guest structural changes induced each other upon inclusion complexation deserve a detailed discussion. The inclusion geometries of various polyphenols containing 3,4-dihydroxybenzene in the β-CD cavity are compared in Table 1 and discussed in Section 2.2.

**Figure 2:**
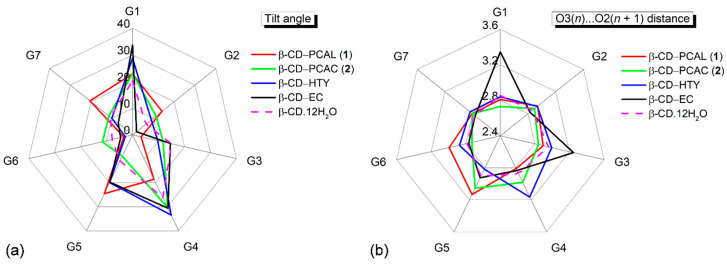
Radar plots comparing (**a**) tilt angles and (**b**) O3(*n*)∙∙∙O2(*n* + 1) distances of the β-CD glucose units (G1–G7) influenced by the inclusion of PCAL (**1**), PCAC (**2**) and HTY [35]. For comparison, data of the inclusion complexes of β-CD–(–)-epicatechin(EC) [43] and the uncomplexed β-CD·12H_2_O [42] are incorporated; see also Table S2. Angles and distances are in ° and Å.

The CD roundness is generally described by (i) the glucose puckering parameters *Q*, *θ* [40] of 0.63 Å, 0° for a perfect ^4^*C*_1_ chair conformation; (ii) the glucose inclination angles < 20° for most glucose units; (iii) the distances related to glycosidic O4, O4(*n*)⋅⋅⋅O4(*n* − 1) and O4(*n*)⋅⋅⋅centroid, describing the well-defined polygon, of which the average of distance ratios is 0.868 for an ideal heptagon; (iv) the endocyclic torsion angles *ϕ*, *ψ* around O4, of which the sum of averages of *ϕ*, *ψ* is close to null [41]; and (v) the O3(*n*)⋅⋅⋅O2(*n* + 1) distances in the short span of 2.770–2.957 Å for the uncomplexed β-CD hydrate [42], see Supplementary Materials, Table S2. The corresponding structural parameters of β-CDs in **1** and **2** are as follows: (i) 0.543(6)–0.592(6) Å, 0.0(4)–9.7(6)°; (ii) 3.3(2)–24.5(2)°, except for G4 (**2**) that has a larger tilt angle, 30.3(2)°; (iii) the average of distance ratios, 0.874–0.876; (iv) the sum of averages, −1.7° and −4.7°; and (v) 2.729(4)–2.992(6) Å, except for G5 (**1** and **2**) that have two outliers of 3.133(6) and 3.056(4) Å (Table S2 and Figure 2). The geometrical parameters of both complexes are comparable to those of β-CD hydrate [42], Table S2. By contrast, β-CD is significantly affected by the encapsulation of bulkier polyphenol, tea (–)-epicatechin (EC) [43], as indicated by the most different structural parameters, two tilt angles of 30.6(1)°, 33.7(1)° and two O3⋅⋅⋅O2 distances of 3.246(3), 3.346(3) Å, Figure 2 and Table S2.

**Figure 3:**
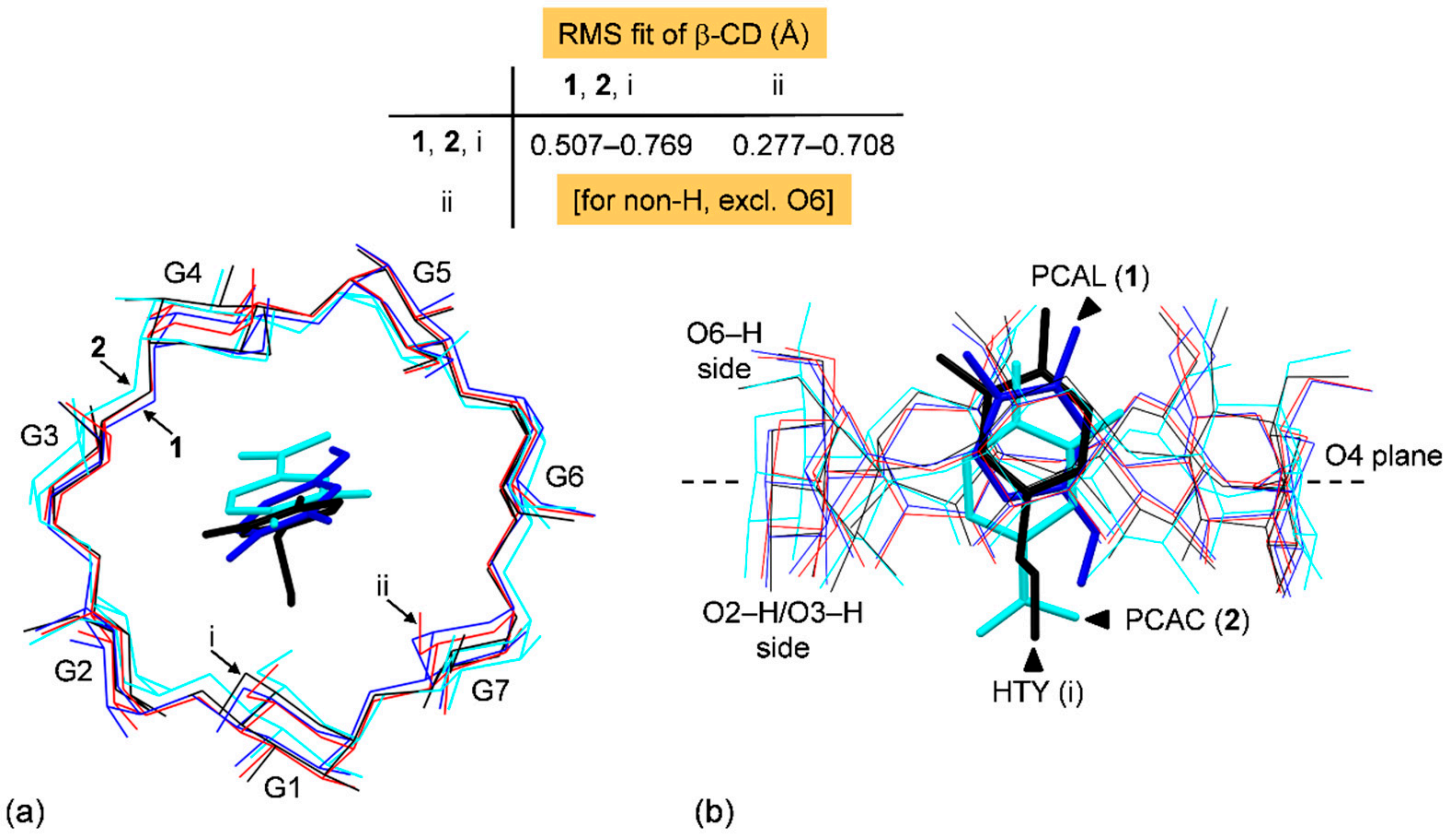
Structure overlays of four β-CDs (blue, cyan, black, and red wireframes, respectively) in complex with PCAL (**1**), PCAC (**2**), HTY (i; [35]), and in the uncomplexed β-CD·12H_2_O (ii; [42]), viewed from (**a**) the top and (**b**) the side. The rms fits are computed for the host β-CDs, disregarding O6, H atoms and guests; see also Table S2.

Overall, the similarity of a CD structure pair is evaluated by the root-mean-square (rms) deviation of superposition as depicted in Figure 3. The β-CD C1–C6 and O2–O5 atoms are included in the rms fit calculation, disregarded O6. The embedded PCAL (**1**), PCAC (**2**) and HTY (labeled i; [35]) affect β-CDs to some extent as indicated by the respective rms fits of 0.277, 0.708 and 0.444 Å, compared to uncomplexed β-CD hydrate (ii; [42]). Among the three complexes, the rms fits are 0.507–0.769 Å, indicating that the complexed β-CDs in the solid state are quite different. This is also reflected in the distinct DFT-derived β-CD molecular energies listed in Table S6.

The H-bonding functionality of the β-CD OH groups affecting its structure should be further noted. Whereas O6–H groups are freely rotatable to make H-bonding to the surrounding water molecules and β-CD OH groups, O2–H/O3–H groups are more restricted due to their engagement in the interglucose flip-flop O3⋅⋅⋅O2 H-bonds [44]. The systematic intramolecular O3⋅⋅⋅O2 H-bonds act like a belt to secure the annular β-CD conformation, thus these OH groups are less available for interaction intermolecularly. The glucose tilt angle and the interglucose O3(*n*)⋅⋅⋅O2(*n* + 1) distances are closely related parameters, as indicated by the similar peak positions of the radar plots (Figure 2a,b). This is notable in the β-CD–EC inclusion complex [43], of which β-CD is more distorted from a round conformation. The large tilt angles of G1 and G4 (33.7(1)° and 30.6(1)°) result in longer O31⋅⋅⋅O22 and O33⋅⋅⋅O24 distances (3.346(3) and 3.246(3) Å), Table S2 and Figure 2a,b. Interestingly, out of seven fully occupied O6–H groups, six (**1**) and four (**2**) groups point outward the β-CD cavity with the exocyclic torsion angles *χ*, *ω* of 45.9(7)–61.9(6)°, −59.4(6)° to −73.7(6)°, Table S2. The remaining four O6–H groups direct inward the β-CD cavity; the corresponding torsion angles are −168.0(3)° to −177.2(3)°, 177.8(3)° and 60.4(4)–70.5(4)°, Table S2. O6–H groups contribute significantly to the lattice stability through making OH⋯O hydrogen bond networks, particularly for the complex β-CD–PCAC (**2**) (Section 2.3, Figure 5, and Tables S3 and S4).

### 2.2. Common Inclusion Geometry of Catechol-Containing Antioxidants

The polyphenolic plant extracts containing an ortho-dihydroxybenzene (catechol) are powerful antioxidant [45] due to the greater molecular stability of intramolecular O–H⋯O H-bond [46] (Figure 1 and Table 2). Principally, on the basis of host–guest space-fit, the opposed 3,4-di(OH) and 1-COOH/1-CHO groups on the aromatic ring of PCAL/PCAC could be placed nearby both β-CD perimeters, giving two equally plausible inclusion modes. However, the belt of O2⋯O3 H-bonds make O2–H, O3–H groups less available and O6–H groups are more ready for H-bonding. Moreover, our previous investigations on the β-CD encapsulation of olive HTY, OLE [35] and coffee CFA, CGA [36] showed that all the guest polyphenols prefer pointing their 3,4-di(OH) groups to the β-CD O6–H side for the energetically stable inclusion complexation. The thermodynamic stabilities of β-CD inclusion complexes with PCAL and PCAC relative to other relevant complexes as inferred from DFT full-geometry optimization are given in Section 2.4. All together, we envisage that both structurally related antioxidants, PCAL and PCAC, are similarly entrapped in the β-CD cavity. The details of inclusion structures in **1** and **2** revealed by X-ray analysis are described below.

**Figure 4:**
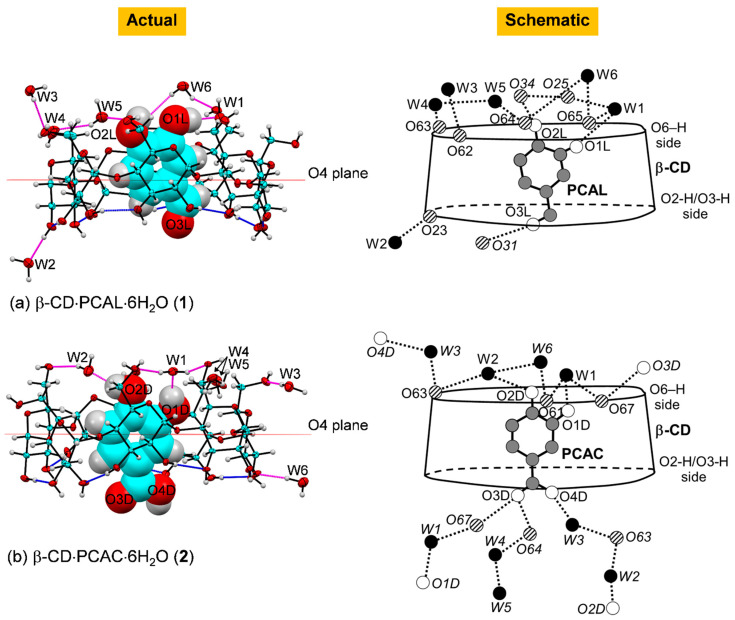
Actual and schematic diagrams depicting inclusion geometries and hydrogen bonding of the complexes (**a**) β-CD–PCAL (**1**) and (**b**) β-CD–PCAC (**2**) in the solid state at 296 K; ORTEP plot at 30% probability level. In actual diagram presenting the asymmetric unit, O–H⋯O hydrogen bonds within β-CD and between other molecules are shown with blue and magenta connecting lines; respectively. In schematic diagram, O–H⋯O hydrogen bonds are shown with dotted lines; names in italics indicate molecules/groups of the adjacent asymmetric units; see also Figure 5 and Table 2.

**Figure 5:**
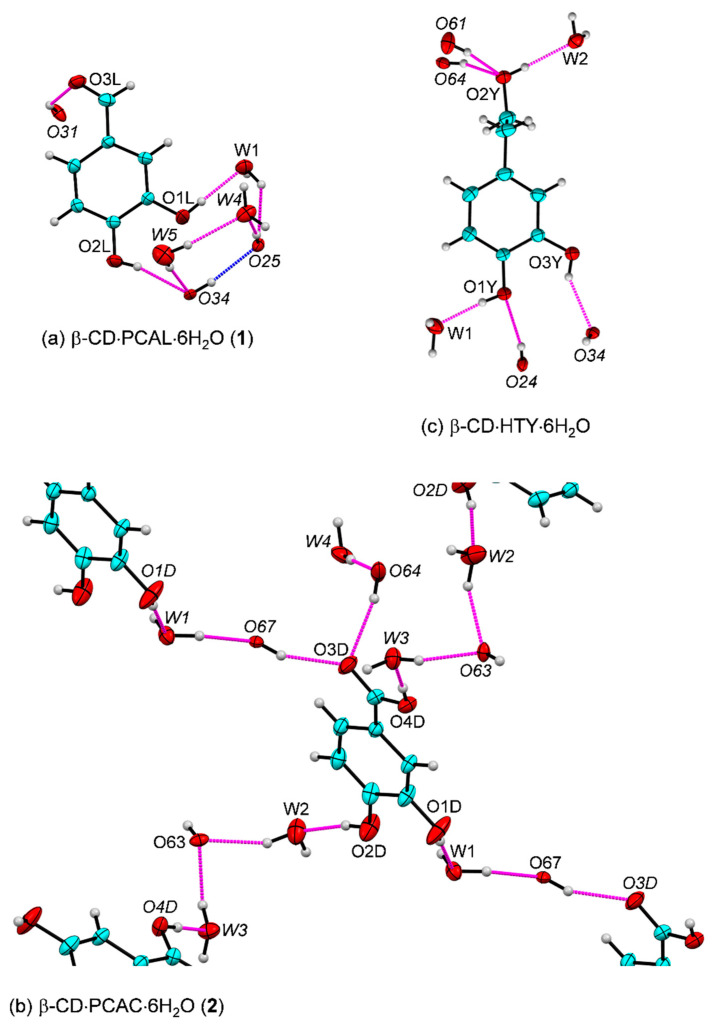
Intermolecular O–H⋯O hydrogen bonds (magenta lines) in the β-CD encapsulation of (**a**) PCAL, **1** and (**b**) PCAC, **2**. (**c**) The inclusion complex of β-CD·HTY·6H_2_O is shown for comparison (adapted from [35]). Names in italics indicate molecules/groups of the adjacent asymmetric units; see also Figure 4 and Table 2. In **1**, the intramolecular O34–H···O25 H-bond stabilizing the round β-CD structure (blue line) is supported by the water network. In **2**, PCAC molecules embedded in the neighboring β-CD cavities are linked via bridging water molecules and β-CD O6–H groups. The β-CD cavities accommodating the PCAL and PCAC molecules are omitted for clarity. The ORTEP diagrams are drawn with 30% probability level.

In the limited space of the β-CD nanocavity, the aromatic guest molecule with para-substituted groups does not rotate freely but prefers aligning the aromatic ring (AR) about a right angle against the β-CD molecular plane (O4 plane). This is the case for PCAL (**1**) and PCAC (**2**), as indicated by the respective interplanar angle of 74.9(1)° and 71.5(1)°, Table 1. Their AR centroids are 0.787 Å above and 0.739 Å beneath the β-CD O4 plane, facilitating 3,4-di(OH) group nearby the O6-side to H-bond with the adjacent water molecules and O3–H group, and CHO, COOH groups around the O2/O3-side to interact with the surrounding O3–H, O6–H and water molecule (Figure 4 and Table 2). This results in O–H⋯O H-bonding network stabilizing the solid structures of **1** and **2** (Figure 5 and Table 2, Tables S3 and S4). The atomic-level inclusion structures of **1** and **2** from single-crystal X-ray diffraction go beyond the overall solution structures derived spectroscopically [27,29].

Moreover, a question rises how **1** and **2** are compared to other polyphenolic complexes and their meanings. Similar inclusion geometries of olive HTY, OLE in the β-CD cavity are observed for the equimolar complexes [35]. However, coffee CFA and CGA with additional CH_2_=CH–C(=O)O and quinic acid (QNA) moieties are bulkier than PCAL and PCAC. This requires an extended space of the β-CD dimeric cavity to optimally anchor CFA and CGA [36]. Certainly, the distinct inclusion structures are observed as only the QNA portion of CGA is outside the wall while CFA is totally embedded in the dimeric cavity, Table 1 [36]. The AR moieties of CFA and CGA are oriented mostly vertically in a larger space of the β-CD dimer interface, Table 1 [36]. Note that the intermolecular OH⋯O H-bonds between o-di(OH) of catechol antioxidants and β-CD OH groups and the shielding of OH groups in the β-CD wall help to improve molecular stability and antioxidant activity in the β-CD cavity [35,36,43]; see Section 2.4.

### 2.3. COOH Group of PCAC Makes the Distinction in Hydrogen Bonding Network

Figure 5 provides a main part of the picture for the solid-state stabilization of **1** and **2**. The monoclinic asymmetric units comprising (C_6_H_10_O_5_)_7_⋅C_6_H_3_(OH)_2_(CHO)⋅6H_2_O (**1**) and (C_6_H_10_O_5_)_7_⋅C_6_H_3_(OH)_2_(COOH)⋅ 6H_2_O (**2**) with a minute difference of H and OH on C7 make a greater distinction in the H-bonding network of both complexes. In **1**, PCAL without OH group on C7, its O3L atom accepts H-bond from β-CD O31–H group and O1L–H, O2L–H groups together with water molecules O1W, O4W, O5W help to secure the intramolecular O34–H⋯O25 H-bond of the β-CD macrocycle (Figure 5a and Table 2, Table S3). In **2**, the extensive complex H-bond network of the adjacent entrapped PCAC molecules as a result from the presence of COOH group is notable. The two H-bond chains linking one PCAC to others are built from bridging water molecules and β-CD O6–H groups: (i) O1D–H⋯O1W–H⋯O67–H⋯O3D; and (ii) O4D–H⋯O3W–H⋯O63⋯H–O2W⋯H–O2D (Figure 5b and Table 2, Table S4).

The well-ordered six water molecules of hydration distributed outside the β-CD cavity, in the intermolecular interstices make H-bonds to the surrounding OH groups of both β-CD rims and to each other. They play a key role as H-bonding mediators in stabilizing the crystal lattice (Table 2, Tables S3 and S4). Note that the PCAL and PCAC 3,4-di(OH) groups (the key moiety contributing to the antioxidant capacity of flavonoids) in **1**, **2** and the β-CD–olive HTY are stabilized through H-bonding to the adjacent β-CD OH groups and water molecules (Figure 5c; [35]). What would happen if the crystal contacts were absent, i.e., the sole host–guest interactions were considered? (see Section 2.4).

### 2.4. Thermodynamic Stabilities of β-CD–PCAL and β-CD–PCAC

A structural chemistry study including DFT complete-geometry optimization and single-crystal X-ray diffraction share several important complementary results. Whereas X-ray analysis provides accurately determined space-and-time average crystal and molecular structures of the crystallizable compounds, computational chemistry (particularly the popular DFT method) gives relevant thermodynamic quantities of molecules and complexes. For the somewhat flexible, large-ring CDs, the available X-ray-derived atomic coordinates were usually used as a starting structure for a better convergence of the DFT energy minimization. The DFT full-geometry optimization in vacuum was adequate to provide meaningful thermodynamic quantities of CD supramolecular complexes in reasonable computing time (especially the stabilization energies). The DFT calculation in implicit solvent took much longer computing time but did not improve the host–guest complexation energies as pointed out in our previous works on the β-CD–tea catechin [43] and β-CD–tricyclic antidepressant [37].

**Figure 6:**
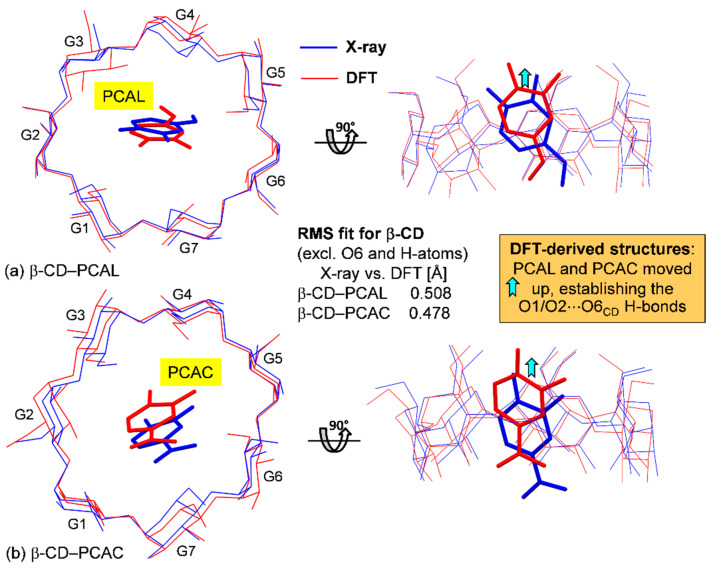
Structural comparison of the inclusion complexes (**a**) β-CD–PCAL and (**b**) β-CD–PCAC from X-ray analysis (blue lines) and DFT calculation in the gas phase (red lines); see also Figure 7, Figure S1 and Tables S5 and S6. The rms fits are calculated for the non-H atoms using the X-ray-derived β-CD skeleton as a reference structure (excluding O6 atoms). Note that after DFT full-geometry optimization, PCAL and PCAC molecules are shifted up to form O1/O2···O6 H-bonds with β-CD O6–H perimeter (Table 2).

Here, the DFT full-geometry optimization in the gas phase converged smoothly. The host β-CD structures in both complexes are changed to some extent after DFT energy minimization but are still superimposable on the original X-ray structures, as indicated by the rms fits of 0.508 Å (β-CD–PCAL) and 0.478 Å (β-CD–PCAC), Figure 6 and Figure S1; the calculations considered only the non-H atoms of β-CD backbone, excluding O6. Moreover, the O2⋯O3 distances of neighboring glucoses in the DFT-derived β-CD structures tend to be lengthened, compared to the original X-ray structure (Tables S3–S5). This is to facilitate the large tilting of glucose units G3 (30.6°) and G2 (27.8°) to better space-fit the rising guest molecules, PCAL and PCAC, respectively (see the cyan arrows in Figure 6). Clearly, this is a paradigm of the well-known induced-fit mechanism [47] necessary for CD inclusion complexes and other biological molecules [48]. In both DFT- and X-ray-derived structures, the comparatively round β-CDs are maintained by systematic interglucose O3⋯O2 H-bonds (Tables S3–S5).

**Figure 7:**
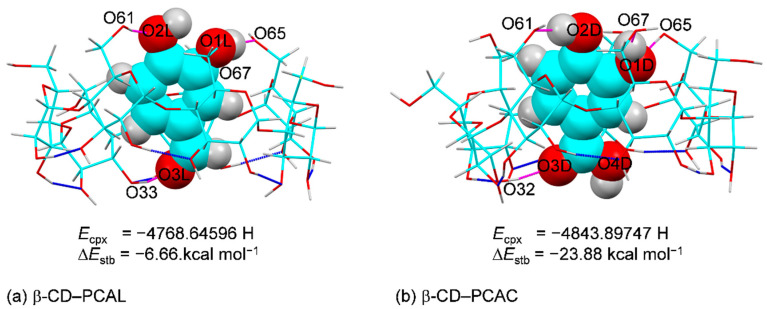
Inclusion complexes of (**a**) β-CD−PCAL and (**b**) β-CD−PCAC derived from DFT full-geometry optimization in the gas phase. For comparison, the energy of complex (*E*_cpx_) and stabilization energy (Δ*E*_stb_) are given; see also Figure S1 and Tables S5 and S6. The intramolecular O3(*n*)∙∙∙O2(*n* + 1) H-bonds of β-CD and host–guest O–H∙∙∙O H-bonds are indicated by blue and magenta connecting lines, respectively.

Whereas the complex energies (*E*_cpx_s) of β-CD–PCAL and β-CD–PCAC cannot be directly compared due to the slight chemical composition differences of the guest molecules, their stabilization and interaction energies (Δ*E*_stb_ and Δ*E*_int_) are applicable. For both complexes, the values of Δ*E*_stb_ and Δ*E*_int_, −6.66 to −23.88 and −12.55 to −30.53 kcal mol^–1^ are comparable to those of β-CD complexes with other polyphenols, olive HTY, OLE [35] and coffee CFA, CGA [36], and fall in a regular energy range for weak non-covalent interactions (Table S6). Note that although both β-CD–PCAL and β-CD–PCAC complexes have the same type and number of host–guest OH⋯O H-bond interactions (4), β-CD–PCAC is 17.22 and 17.98 kcal mol^–1^ more stable than β-CD–PCAL, based on ΔΔ*E*_stb_ and ΔΔ*E*_int_ (Tables S5 and S6). This is due mainly to β-CD of β-CD–PCAC is less stable (more deformed) than that of β-CD–PCAL by 13.72 and 13.28 kcal mol^–1^, based on their energy differences, ΔΔ*E*_β-CD___opt_ and ΔΔ*E*_β-CD___sp_ (Table S6). Moreover, the DFT-optimized structures of both complexes in the gas phase have ascending shifts of PCAL and PCAC to the β-CD O6–H-side for energetically stable inclusion geometries, which are maintained by four intermolecular OH⋯O H-bonds on both β-CD rims. These four newly formed host–guest OH⋯O H-bonds are present in the gas phase to compensate the thermodynamic stability lose due to the extinction of crystal contacts (Figure 7, Figure S1 and Table 2, Tables S5 and S6). However, the contrary is observed in solution. β-CD–PCAL is at least one order of magnitude more stable than β-CD–PCAC, as indicated by the respective binding constants of 6700 ± 777 M^–1^ (UV-vis) [29] and 264 ± 29 M^−1^ (UV-vis) [28], 661 M^−1^ (NMR) [27].

Among various β-CD–polyphenol complexes (Table S6), the complex with olive OLE is most stable due to the greater number of host–guest OH⋯O H-bonds (6). By contrast, the β-CD–tricyclic antidepressant (TCA) complexes with the aromatic moiety entrapped and primarily kept in position by CH⋯π interactions [37,38]. The corresponding Δ*E*_stb_ and Δ*E*_int_ about −4 to −8 kcal mol^–1^ of the TCA complexes indicate that they are ~2–4 times less stable than the H-bond-stabilized complexes of polyphenols.

### 2.5. Bioactive PCAL and PCAC Are Rather Rigid in Distinct Lattice Circumstances

**Figure 8:**
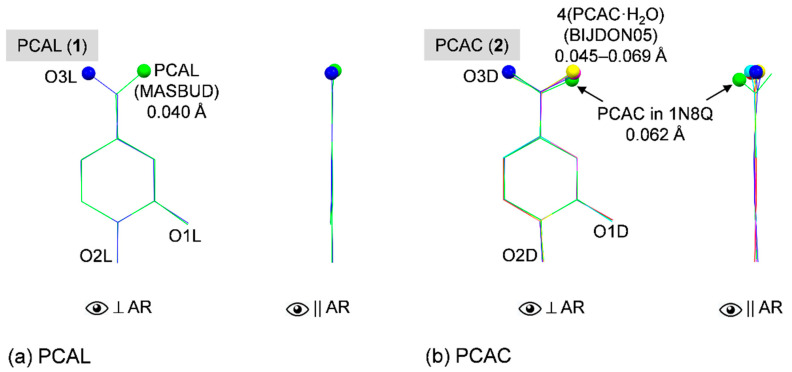
Structure overlays of (**a**) PCAL and (**b**) PCAC in uncomplexed form, in β-CD cavity and in complex with protein (if available), viewed perpendicular and parallel to the aromatic ring (AR). Reference structures are shown in blue wireframes of which their names are marked in gray areas and only non-H atoms of the rigid parts of polyphenols (excluding O3 and O4) are used for calculating the rms fits. The corresponding rms fit for each structure pair is indicated by nearby distance. Note that the orientation of C7=O3 group with respect to O1 is emphasized by ball model.

Theoretically, the induced-fit mechanism [47] of the CD inclusion complexation works such that both host and guest molecules adapt their structures to some extent to attain a thermodynamically stable adduct. We point out in Section 2.1 that the complexed β-CDs in **1** and **2** are distorted to an extent from a round conformation of the uncomplexed β-CD·12H_2_O [42], as indicated by the respective rms fits of 0.277 and 0.708 Å (Section 2.1). However, a reverse situation is observed for the guest PCAL and PCAC molecules as clearly depicted in Figure 8a,b.

To gain further insight into structural changes of the bioactive PCAL and PCAC, we made a survey through two crystal data banks of small molecules at the Cambridge Crystallographic Data Center (CCDC; www.ccdc.cam.ac.uk; [49]) and of biological molecules at the RCSB Protein Data Bank (RCSB PDB; www.rcsb.org; [50]). The CCDC database search revealed that there is one structure of free PCAL anhydrate (code MASBUD; [51] and two polymorphs of PCAC·H_2_O in the triclinic system (code BIJDON03; [52]; code BIJDON05; [53]) and monoclinic system (code BIJDON04; [54]). Surprisingly, there is just one published data set available in the PDB for PCAC in complex with lipoxygenase (Lox) (code 1N8Q; [55]). Loxs are nonheme iron-containing enzymes for catalysis of lipid deoxygenation, producing the unsaturated fatty acid metabolites, which influence inflammatory diseases and cancer progression. PCAC is a degradation product found nearby the iron site in the soy Lox–quercetin complex [55]. Quercetin in alkaline solution is degraded to PCAC, which is significantly decreased when the catechol moiety of quercetin is embedded in various β-CD cavities [56].

Figure 8a displays the perfect overlay of PCAL in the β-CD cavity and uncomplexed PCAL [51], as indicated by the small rms fit of 0.040 Å, excluded the rotatable O3L (flipped to other side, see balls). CHO groups are in the same plane of the aromatic ring with the small rms deviations of least squares plane of 0.013 and 0.024 Å for the embedded and free PCAL, respectively. PCAC·H_2_O in the triclinic and monoclinic modifications [52,53,54] are isostructural and their COOH groups are coplanar with the aromatic ring because these COOH groups are engaged in the intermolecular OH⋯O H-bonds of the planar PCAC dimer. The planarities of four PCAC molecules in the triclinic asymmetric unit are indicated by the small rms deviations of least squares plane of 0.016–0.035 Å. By contrast, PCAC in complex with β-CD and with Lox [55] allow their COOH groups to deviate from the aromatic plane although their 3,4-dihydroxybenze structures are mostly identical; rms fit = 0.062 Å (Figure 8b). The twisting of the COOH group from the PCAC molecular plane facilitates the H-bonding network in the lattice of the β-CD–PCAC complex (Figure 5b) and the better binding of PCAC in the protein active site [55]. The corresponding rms deviations of least squares plane are 0.051 and 0.221 Å.

## 3. Materials and Methods

### 3.1. Materials

β-CD (>95%) as white solid powder was obtained from Cyclolab, Budapest, Hungary (code CY-2001). PCAL (97%) as tan solid powder was supplied by Sigma-Aldrich, St. Louis, MO, USA, (code D108405). PCAC (97%) as brown crystalline powder was purchased from Acros Organics (code A11489). All chemicals were used as received. The ultrapure water was provided by a Milli-Q Water System.

### 3.2. Single Crystal X-ray Diffraction Analysis

#### 3.2.1. Crystallization

In each separate 1.5-mL vial, the 1:2 solid mixtures of β-CD–PCAL (**1**) and β-CD–PCAC (**2**) prepared from β-CD 50 mg (0.044 mmol), PCAL 12 mg (0.088 mmol) and PCAC 14 mg (0.088 mmol) were dissolved in pure water 1 mL. After heating at 323 K for three hours in an ultrasonic bath, light yellow concentrated solutions of **1** and **2** were obtained. Then the two vials containing solutions were left undisturbed in an air-conditioned room (298 K). Slow solvent evaporation took place for two weeks, single crystals were harvested.

#### 3.2.2. Diffraction Data Collection

Colorless platelet single crystals with dimensions of 0.02 × 0.14 × 0.40 mm (**1**) and 0.02 × 0.24 × 0.32 mm (**2**) each was mounted on the tip of a MiTeGen microloop. X-ray diffraction experiment was conducted at 296(2) K on a Bruker APEXII Kappa CCD diffractometer operated at 50 kV, 30 mA, producing monochromatic MoK_α_ radiation (*λ* = 0.71073 Å). With the help of the APEX2 software suite [57], diffraction image of 600 and 680 frames were collected in *ω*−*ϕ* mode to 0.70 Å atomic resolution with exposure times and scan angles of 8 sec, 1.2° and 10 sec, 1.2° for respective complexes **1** and **2**. Data processing was carried out with standard programs implemented in the APEX2 software suite [57]. The procedures began from data image integration with SAINT [58], followed by data reduction and scaling together with multiscan absorption corrections using SADABS [57] and completed with data merging using XPREP [58], yielding relatively good room-temperature diffraction data with mostly 100% completeness; see the summary below. Details of data statistics are given in Supplementary Materials, Table S1.

#### 3.2.3. Structure Solution and Refinement

The crystal structures of **1** and **2** were solved by intrinsic phasing method with SHELXTL XT [57], providing all non-H atoms of host β-CD, guest PCAL, PCAC, and water molecules. The structures were refined anisotropically by full matrix least squares on *F*^2^ with SHELXTL XLMP [57]. All H-atom positions (excluding those of flexible OH groups and water molecules) were calculated geometrically and treated with a riding model: C−H = 0.95 Å, *U*_iso_ = 1.2*U*_eq_(C)(aromatic); C−H = 1.00 Å, *U*_iso_ = 1.2*U*_eq_(C)(methine); and C−H = 0.99 Å, *U*_iso_ = 1.2*U*_eq_(C)(methylene). The H atoms of β-CD OH groups and six water molecules were initially located from difference Fourier electron density maps. Then the OH H-atoms were refined using ‘AFIX 147′ or ‘AFIX 83′ with restraints O−H = 0.84 Å, *U*_iso_ = 1.5*U*_eq_(O). The two H-atoms of a water molecule were refined with DFIX restraints to idealized geometry (O−H 0.96 Å and H···H 1.52 Å) and with ‘AFIX 3′ constraint *U*_iso_ = 1.5*U*_eq_(water). In the refinement course, BUMP antibumping restraints were applied to prevent short intermolecular H···H distances. The refinement converged nicely to final *R*-factors ~6%; see the summary below and Table S1 for more details.

Note that no disordered atom was found in both β-CD⋅PCAL⋅6H_2_O (**1**) and β-CD⋅PCAC⋅6H_2_O (**2**), i.e., all seven O6-H groups of β-CD, PCAL/PCAC and six water molecules for each complex were fully occupied. The well-ordered structures of **1** and **2** were not frequently observed for the room-temperature diffraction data of CD inclusion complexes [49]. Here, the well-established H-bonding networks facilitate the full order of both complexes, see Section 2.3.

Crystal data for **1** [(C_6_H_10_O_5_)_7_⋅C_7_H_6_O_3_⋅6H_2_O, M = 1381.19 g mol^–1^]: monoclinic, space group *P*2_1_ (no. 4), *a* = 15.4213(11) Å, *b* = 10.0440(7) Å, *c* = 21.2795(15) Å, *β* = 110.281(2)°, *V* = 3091.7(4) Å^3^, *Z* = 2, *T* = 296(2) K, *μ*(MoK_α_) = 0.133 mm^–1^, *D*_calc_ = 1.484 g cm^–3^, 60310 reflections measured (1.41° ≤ *Θ* ≤ 30.67°), 18989 unique (*R*_int_ = 0.0809, *R*_sigma_ = 0.1641) which were used in all calculations. The final *R*_1_ was 0.0620 (*I* > 2*σ*(*I*)) and *wR*_2_ was 0.1527 (all data).

Crystal data for **2** [(C_6_H_10_O_5_)_7_⋅C_7_H_6_O_4_⋅6H_2_O, M = 1397.19 g mol^–1^]: monoclinic, space group *P*2_1_ (no. 4), *a* = 12.5165(7) Å, *b* = 19.0287(12) Å, *c* = 14.0626(8) Å, *β* = 109.656(2)°, *V* = 3154.2(3) Å^3^, *Z* = 2, *T* = 296(2) K, *μ*(MoK_α_) = 0.132 mm^–1^, *D*_calc_ = 1.471 g cm^–3^, 55384 reflections measured (1.54° ≤ *Θ* ≤ 30.61°), 19,288 unique (*R*_int_ = 0.0990, *R*_sigma_ = 0.1339), which were used in all calculations. The final *R*_1_ was 0.0602 (*I* > 2*σ*(*I*)) and *wR*_2_ was 0.1134 (all data).

### 3.3. DFT Full-Geometry Optimization

Meaningful, reliable structures and thermodynamic data of CD inclusion complexes can be deduced from complete-geometry optimization by the DFT method. This has been successfully demonstrated in our previous works on the β-CD encapsulation of polyphenolic plants extracts, including tea epicatechins [59], olive oil [35], coffee chlorogenic and caffeic acids [36] and of tricyclic antidepressant drugs [37,38,39], which are inspired by the DFT calculation of large carbohydrate CA-26 [60].

Starting atomic coordinates of the 1:1 β-CD–PCAL and β-CD–PCAC inclusion complexes were taken from the converged crystal structure refinements of **1** and **2**, excluding the hydration water molecules. Before the calculation, all the underestimated X-ray-derived C–H and O–H distances were normalized to neutron hydrogen distances, 1.083 and 0.983 Å [61]. The two corrected structures were optimized by semiempirical PM3 method and then fully re-optimized by DFT calculation in the gas phase using the B3LYP functional with mixed basis sets 6-31+G* for H, O and 4-31G for C. All calculations were carried out using program GAUSSIAN09 [62] on a DELL PowerEdge T430 server.

Regarding the protonated form of PCAC that was used in the DFT full-geometry optimization of the β-CD–PCAC inclusion complex, PCAC with pKa ~4.5 in a neutral pH solution containing β-CD was expected to exist in an anionic form. However, it turned out that PCAC and β-CD co-crystallized to yield a thermodynamically stable adduct in the single-crystalline state. Similarly, benzoic acid with pKa = 4.2 complexed with β-CD in solution [63] and the β-CD–benzoic acid complex existed in two crystal forms [64,65]. In the crystal structure of the β-CD–PCAC complex, the bond distances C7D=O3D, 1.226(6) Å and C7D–O4D, 1.307(6) Å indicated the structure of benzoic group for net charge balance of the stable complex. Therefore, to mimic the β-CD–PCAC complex in the solid state and to evaluate the inherent thermodynamic stabilization, we selectively adopted the X-ray-derived molecular complex with PCAC in a neutral benzoic acid not in a benzoate form for energy minimization in vacuum by DFT method. After DFT calculation, PCAC was stabilized in the β-CD cavity and its original neutral form retained with corresponding bond distances of 1.235 and 1.359 Å; see Section 2.3.

The stabilization energy and interaction energy of the complex (Δ*E*_stb_ and Δ*E*_int_) were calculated straightforwardly. For example, Δ*E*_stb_ was obtained by subtracting the molecular energies of β-CD and PCAL/PCAC from the energy of the β-CD–PCAL/PCAC complex from the full-geometry optimization. Similarly, Δ*E*_int_ considered the corresponding single-point energies in the complexed states. The DFT-derived inclusion structures of the two complexes together with their O−H⋯O hydrogen bonds, Δ*E*_stb_ and Δ*E*_int_ are given in Supplementary Materials, Figure S1 and Tables S5 and S6.

Note that no basis set superposition error (BSSE) correction is applied to the DFT-derived energies of the β-CD–catechol antioxidant complexes listed in Table S6. This is because the estimated energy differences (ΔΔ*E*_stb_ and ΔΔ*E*_int_) are sufficient to interpret the relative thermodynamic stabilities in relation to host–guest interactions and antioxidant properties.

## 4. Conclusions

Protocatechuic aldehyde (PCAL) and protocatechuic acid (PCAC) are polyphenol extracts from plants. They contain a catechol moiety and thus having wide therapeutic effects including antioxidant, anti-inflammatory, and anticancer activities. Their physicochemical and pharmacological properties have been improved via cyclodextrin (CD) inclusion complexation, of which their characteristics are still controversial. To address at the atomic-level of the inclusion complexation, we investigate the β-CD encapsulation of PCAL (**1**) and PCAC (**2**) by a combined study of single-crystal X-ray diffraction and density functional theory (DFT) full-geometry optimization.

X-ray analysis disclosed that PCAL and PCAC are nearly totally shielded in the β-CD wall. Their aromatic rings are vertically aligned in the β-CD cavity such that the functional groups on the opposite side of the ring (3,4-di(OH) and 1-CHO/1-COOH groups) are placed nearby the O6–H and O2–H/O3–H rims, respectively. The preferred inclusion modes in **1** and **2** help to establish crystal contacts of OH⋅⋅⋅O H-bonds with the adjacent β-CD OH groups and water molecules, which are similar to our previous works on β-CD inclusion complexes with olive HTY and OLE [35]. By contrast, the DFT-optimized structures in the gas phase of both complexes are thermodynamically stable via the four newly formed host–guest OH⋅⋅⋅O H-bonds. The intermolecular OH⋅⋅⋅O H-bonds between PCAL/PCAC 3,4-di(OH) and β-CD OH groups and the shielding of OH groups in the β-CD wall help to stabilize these antioxidants in the β-CD cavity, as observed in our earlier studies. Moreover, PCAL and PCAC bearing CHO and COOH groups in distinct lattice environments (uncomplexed form, confined in β-CD cavity, and in complex with protein) are compared, revealing their structural rigidity. An atomistic perspective of the β-CD encapsulation of catechol antioxidants PCAL and PCAC through the lens of X-ray crystallography and DFT calculation goes beyond the spectroscopically derived inclusion structures in solution.

## Figures and Tables

**Table 1:** Inclusion Structures of PCAL (**1**), PCAC (**2**) Encapsulated in the β-CD Cavity, in Comparison to Other 1:1 β-CD–3,4-Dihydroxybenzene Complexes ^a^.

	PCAL (1)	PCAC (2)	HTY ^b^	OLE ^b^	CFA ^c^	CGA ^c^
AR vs. β-CD-O4 interplanar angle [°] ^d^	74.9(1)	71.5(1)	85.0(1)	59.2	87.9(7)	89.3(1)
Moiety nearby/protruded from β-CD O6-side	OH	OH	OH	OH	COOH	QNA
Moiety enclosed in β-CD cavity	AR	AR	AR	AR	C=C−COOH	C=C−COO
No. of free OH ^e^						
In each polyphenol	2	3	3	6	3	6
Shielded in β-CD [dimeric] cavity	2	3	0	0	[3]	[6]
Distance from guest to β-CD [Å]						
AR centroid to O4 centroid (diagonal) ^f^	0.837	−0.779	0.990	1.016	−3.987	−4.450
AR centroid to O4 plane (vertical)	0.787	−0.739	0.986	0.871	−3.453	−4.182

^a^ Polyphenols with catechol (ortho-dihydroxybenzene) scaffold including hydroxytyrosol (HTY), oleuropein (OLE) in olive, and chlorogenic acid (CGA), a conjugate of caffeic acid (CFA) and quinic acid (QNA), CFA in coffee. ^b^ One HTY and one OLE molecule in the β-CD cavity [35]. ^c^ One CFA and two CGA molecules in the β-CD dimeric cavity [36]. ^d^ Interplanar angle between polyphenol aromatic ring (AR) and CD molecular plane (common O4 mean plane). ^e^ Free OH including OH of COOH that are available for intermolecular H-bonding. ^f^ When the CD O6-side pointing upwards, the positive(negative) values indicate that the AR centroid of guest is above(beneath) the host molecular plane.

**Table 2:** O−H⋅⋅⋅O Hydrogen Bonds Involving PCAL and PCAC from X-ray Analysis and DFT Calculation [Å, ^o^]. ^a.^

Interaction	O−H	O⋅⋅⋅O	∠(OHO)	Interaction	O−H	O⋅⋅⋅O	∠(OHO)
X-ray				DFT ^c^			
β-CD⋅PCAL (1)				β-CD⋅PCAL ^d^			
O2L−H⋅⋅⋅O1L	0.82	2.700(6)	114.1	O2L−H⋅⋅⋅O1L	0.98	2.69	114.4
O1L−H⋅⋅⋅O1W	0.82	2.658(6)	177.7	O61−H⋅⋅⋅O2L	0.97	2.95	164.7
O2L−H⋅⋅⋅O34 ^i b^	0.82	2.874(6)	166.0	O33−H⋅⋅⋅O3L	0.98	2.93	164.7
O31−H⋅⋅⋅O3L ^ii^	0.82	2.842(6)	134.4	O67−H⋅⋅⋅O1L	0.97	3.02	169.3
				O1L−H⋅⋅⋅O65	0.99	2.74	177.8
β-CD⋅PCAC (2)				β-CD⋅PCAC ^d^			
O1D−H⋅⋅⋅O2D	0.82	2.703(5)	108.7	O1D−H⋅⋅⋅O2D	0.99	2.69	108.7
O1D−H⋅⋅⋅O1W	0.82	2.705(5)	168.9	O65−H⋅⋅⋅O1D	0.97	3.02	177.5
O2D−H⋅⋅⋅O2W	0.82	2.670(6)	167.3	O1D−H⋅⋅⋅O67	0.99	3.01	145.1
O64−H⋅⋅⋅O3D ^iii^	0.82	2.937(6)	167.4	O2D−H⋅⋅⋅O61	0.99	2.78	161.9
O67−H⋅⋅⋅O3D ^iv^	0.82	2.767(5)	154.3	O32−H⋅⋅⋅O3D	0.98	2.90	169.4
O4D−H⋅⋅⋅O3W ^v^	0.82	2.631(5)	171.5				

^a^ Complete lists of O−H⋅⋅⋅O H-bond interactions are given in Tables S3 and S4 (X-ray) and S5 (DFT). ^b^ Equivalent positions: (i) *x*, *y* − 1, *z*; (ii) −*x* + 1, *y* − 0.5, −*z* + 2; (iii) −*x* + 2, *y* − 0.5, −*z* + 1; (iv) *x* − 1, *y*, *z*; (v) *x* + 1, *y*, *z*. ^c^ DFT calculation in vacuum at B3LYP/6-31+G*/4-31G level, see also Figure S1 and Table S6. ^d^ X-ray-derived structures were used as starting models.

## Data Availability

The data are contained within the article. Additional crystallographic and computational data are available in Supplementary Materials and the Cambridge Crystallographic Data Centre (CCDC).

## Supplementary Materials

The following are available online. Figure S1: Inclusion complexes of (a) β-CD−PCAL and (b) β-CD−PCAC, derived from DFT complete-geometry optimization in the gas phase, Table S1: X-ray single crystal data collection and refinement statistics of 1 and 2, Table S2: Comparison of β-CD geometrical parameters in 1, 2, β-CD⋅12H2O and β-CD⋅(–)-epicatechin⋅4.2H2O, Table S3: OH⋯O hydrogen bonds in β-CD⋅PCAL⋅6H2O (1), Table S4: OH⋯O hydrogen bonds in β-CD⋅PCAC⋅6H2O (2), Table S5: OH⋯O hydrogen bonds in β-CD–PCAL and β-CD–PCAC inclusion complexes from DFT full-geometry optimization, Table S6: Stabilization and interaction energies of β-CD–PCAL and β-CD–PCAC complexes compared to other β-CD–3,4-dihydroxybenzene complexes from DFT full-geometry optimization. Crystallographic data of 1 and 2 have been deposited at the Cambridge Crystallographic Data Centre (CCDC) under respective reference numbers 2075723 and 2075722. Author have read and agreed to the published version of the manuscript.

## References

[1] Rababah T.M., Hettiarachchy N.S., Horax R. (2004). Total phenolics and antioxidant activities of fenugreek, green tea, black tea, grape seed, ginger, rosemary, gotu kola, and ginkgo extracts, vitamin E, and tert-butylhydroquinone. J. Agric. Food Chem..

[2] Pacheco-Palencia L.A., Mertens-Talcott S., Talcott S.T. (2008). Chemical composition, antioxidant properties, and thermal stability of a phytochemical enriched oil from Acai (*Euterpe oleracea* Mart.). J. Agric. Food Chem..

[3] Chao C.Y., Yin M.C. (2009). Antibacterial effects of roselle calyx extracts and protocatechuic acid in ground beef and apple juice. Foodborne Pathog. Dis..

[4] Palacios I., Lozano M., Moro C., D’arrigo M., Rostagno M.A., Martínez J.A., García-Lafuente A., Guillamón E., Villares A. (2011). Antioxidant properties of phenolic compounds occurring in edible mushrooms. Food Chem..

[5] Vitaglione P., Donnarumma G., Napolitano A., Galvano F., Gallo A., Scalfi L., Fogliano V. (2007). Protocatechuic acid is the major human metabolite of cyanidin-glucosides. J. Nutr..

[6] Lee Y.S., Kang Y.H., Jung J.Y., Lee S., Ohuchi K., Shin K.H., Kang I.J., Park J.H., Shin H.K., Lim S.S. (2008). Protein glycation inhibitors from the fruiting body of *Phellinus linteus*. Biol. Pharm. Bull..

[7] Zhou Z., Liu Y., Miao A.D., Wang S.Q. (2005). Protocatechuic aldehyde suppresses TNF-α-induced ICAM-1 and VCAM-1 expression in human umbilical vein endothelial cells. Eur. J. Pharmacol..

[8] Etoh H., Murakami K., Yogoh T., Ishikawa H., Fukuyama Y., Tanaka H. (2004). Anti-oxidative compounds in barley tea. Biosci. Biotechnol. Biochem..

[9] Xu M., Zhang Z., Fu G., Sun S., Sun J., Yang M., Liu A., Han J., Guo D. (2007). Liquid chromatography–tandem mass spectrometry analysis of protocatechuic aldehyde and its phase I and II metabolites in rat. J. Chromatogr. B.

[10] Conde E., Cadahía E., García-Vallejo M.C., Fernández de Simón B. (1998). Polyphenolic composition of *Quercus suber* cork from different Spanish provenances. J. Agric. Food Chem..

[11] Masella R., Santangelo C., D’archivio M., LiVolti G., Giovannini C., Galvano F. (2012). Protocatechuic acid and human disease prevention: Biological activities and molecular mechanisms. Curr. Med. Chem..

[12] Kakkar S., Bais S. (2014). A review on protocatechuic acid and its pharmacological potential. ISRN Pharmacol..

[13] Yan X. (2014). Dan Shen (Salvia miltiorrhiza) in Medicine.

[14] Semaming Y., Pannengpetch P., Chattipakorn S.C., Chattipakorn N. (2015). Pharmacological properties of protocatechuic acid and its potential roles as complementary medicine. Evid Based Compl. Alt. Med..

[15] Khan A.K., Rashid R., Fatima N., Mahmood S., Mir S., Khan S., Jabeen N., Murtaza G. (2015). Pharmacological activities of protocatechuic acid. Acta Pol. Pharm..

[16] Song J., He Y., Luo C., Feng B., Ran F., Xu H., Cia Z., Xua R., Han L., Zhang D. (2020). New progress in the pharmacology of protocatechuic acid: A compound ingested in daily foods and herbs frequently and heavily. Pharmacol. Res..

[17] Chang Z.Q., Gebru E., Lee S.P., Rhee M.H., Kim J.C., Cheng H., Park S.C. (2011). In vitro antioxidant and anti-inflammatory activities of protocatechualdehyde isolated from *Phellinus gilvus*. J. Nutr. Sci. Vitaminol..

[18] Jeong J.B., Lee S.H. (2013). Protocatechualdehyde possesses anti-cancer activity through downregulating cyclin D1 and HDAC2 in human colorectal cancer cells. Biochem. Biophys. Res. Commun..

[19] Wang B.Q. (2010). Salvia miltiorrhiza: Chemical and pharmacological review of a medicinal plant. J. Med. Plant. Res..

[20] Wang L., Ma R., Liu C., Liu H., Zhu R., Guo S., Tang M., Li Y., Niu J., Fu M. (2017). Salvia miltiorrhiza: A potential red light to the development of cardiovascular diseases. Curr. Pharm. Des..

[21] Sroka Z., Cisowski W. (2003). Hydrogen peroxide scavenging, antioxidant and anti-radical activity of some phenolic acids. Food Chem. Toxicol..

[22] Li X., Wang X., Chen D., Chen S. (2011). Antioxidant activity and mechanism of protocatechuic acid in vitro. Funct. Food Health Dis..

[23] Saito S., Kawabata J. (2006). DPPH (= 2, 2-diphenyl-1-picrylhydrazyl) radical-scavenging reaction of protocatechuic acid (= 3, 4-dihydroxybenzoic acid): Difference in reactivity between acids and their esters. Helv. Chim. Acta.

[24] Saenger W. (1980). Cyclodextrin inclusion compounds in research and industry. Angew. Chem. Int. Ed. Engl..

[25] Dodziuk H. (2006). Cyclodextrins and Their Complexes: Chemistry, Analytical Methods, Applications.

[26] Pinho E., Grootveld M., Soares G., Henriques M. (2014). Cyclodextrins as encapsulation agents for plant bioactive compounds. Carbohydr. Polym..

[27] Rescifina A., Chiacchio U., Iannazzo D., Piperno A., Romeo G. (2010). β-cyclodextrin and caffeine complexes with natural polyphenols from olive and olive oils: NMR, thermodynamic, and molecular modeling studies. J. Agric. Food Chem..

[28] Irwin P.L., Pfeffer P.E., Doner L.W., Sapers G.M., Brewster J.D., Nagahashi G., Hicks K.B. (1994). Binding geometry, stoichiometry, and thermodynamics of cyclomalto-oligosaccharide (cyclodextrin) inclusion complex formation with chlorogenic acid, the major substrate of apple polyphenol oxidase. Carbohydr. Res..

[29] Divakar S., Maheswaran M.M. (1997). Structural studies on inclusion compounds of β-cyclodextrin with some substituted phenols. J. Incl. Phenom. Macrocycl. Chem..

[30] Cao J., Wei J., Tian K., Su H., Wan J., Li P. (2014). Simultaneous determination of seven phenolic acids in three *Salvia* species by capillary zone electrophoresis with β-cyclodextrin as modifier. J. Sep. Sci..

[31] Mourtzinos I., Makris D.P., Yannakopoulou K., Kalogeropoulos N., Michali I., Karathanos V.T. (2008). Thermal stability of anthocyanin extract of Hibiscus sabdariffa L. in the presence of β-cyclodextrin. J. Agric. Food Chem..

[32] Kalogeropoulos N., Konteles S., Mourtzinos I., Troullidou E., Chiou A., Karathanos V.T. (2009). Encapsulation of complex extracts in β-cyclodextrin: An application to propolis ethanolic extract. J. Microencapsul..

[33] Kalogeropoulos N., Yannakopoulou K., Gioxari A., Chiou A., Makris D.P. (2010). Polyphenol characterization and encapsulation in β-cyclodextrin of a flavonoid-rich Hypericum perforatum (St John’s wort) extract. LWT Food Sci. Technol..

[34] Ibrahim S., Bowra S. (2019). Improving Oxidative Stability of Polyphenolic Fraction of Apple Pomace by Encapsulation Using Naturally Occurring Polymers. J. Encapsul. Adsorp. Sci..

[35] Aree T., Jongrungruangchok S. (2018). Structure–antioxidant activity relationship of β-cyclodextrin inclusion complexes with olive tyrosol, hydroxytyrosol and oleuropein: Deep insights from X-ray analysis, DFT calculation and DPPH assay. Carbohydr. Polym..

[36] Aree T. (2019). Understanding structures and thermodynamics of β-cyclodextrin encapsulation of chlorogenic, caffeic and quinic acids: Implications for enriching antioxidant capacity and masking bitterness in coffee. Food Chem..

[37] Aree T. (2020). β-Cyclodextrin encapsulation of nortriptyline HCl and amitriptyline HCl: Molecular insights from single-crystal X-ray diffraction and DFT calculation. Int. J. Pharm..

[38] Aree T. (2020). β-Cyclodextrin inclusion complexation with tricyclic antidepressants desipramine and imipramine: A structural chemistry perspective. J. Pharm. Sci..

[39] Aree T. (2020). Supramolecular Complexes of β-Cyclodextrin with Clomipramine and Doxepin: Effect of the Ring Substituent and Component of Drugs on Their Inclusion Topologies and Structural Flexibilities. Pharmaceuticals.

[40] Cremer D.T., Pople J.A. (1975). General definition of ring puckering coordinates. J. Am. Chem. Soc..

[41] French A.D., Johnson G.P. (2007). Linkage and pyranosyl ring twisting in cyclodextrins. Carbohydr. Res..

[42] Lindner K., Saenger W. (1982). Crystal and molecular structure of cyclohepta-amylose dodecahydrate. Carbohydr. Res..

[43] Aree T., Jongrungruangchok S. (2016). Crystallographic evidence for β-cyclodextrin inclusion complexation facilitating the improvement of antioxidant activity of tea (+)-catechin and (−)-epicatechin. Carbohydr. Polym..

[44] Saenger W., Betzel C., Hingerty B., Brown G.M. (1982). Flip-flop hydrogen bonding in a partially disordered system. Nature.

[45] Terao J. (2009). Dietary flavonoids as antioxidants. Food Fact. Health Promot..

[46] Lucarini M., Pedulli G.F., Guerra M. (2004). A Critical Evaluation of the Factors Determining the Effect of Intramolecular Hydrogen Bonding on the O–H Bond Dissociation Enthalpy of Catechol and of Flavonoid Antioxidants. Chem. Eur. J..

[47] Koshland D.E. (1973). Protein shape and biological control. Sci. Am..

[48] Jeffrey G.A., Saenger W. (2012). Hydrogen Bonding in Biological Structures.

[49] Groom C.R., Bruno I.J., Lightfoot M.P., Ward S.C. (2016). The Cambridge structural database. Acta Cryst..

[50] Rose P.W., Prlić A., Bi C., Bluhm W.F., Christie C.H., Dutta S., Green R.K., Goodsell D.S., Westbrook J.D., Woo J. (2015). The RCSB Protein Data Bank: Views of structural biology for basic and applied research and education. Nucleic Acids Res..

[51] Sarma J.A., Nagaraju A., Majumdar K.K., Samuel P.M., Das I., Roy S., McGhie A.J. (2000). Solid state nuclear bromination with N-bromosuccinimide. Part 2. Experimental and theoretical studies of reactions with some substituted benzaldehydes. J. Chem. Soc. Perkin Trans..

[52] Horneffer V., Dreisewerd K., Lüdemann H.C., Hillenkamp F., Läge M., Strupat K. (1999). Is the incorporation of analytes into matrix crystals a prerequisite for matrix-assisted laser desorption/ionization mass spectrometry? A study of five positional isomers of dihydroxybenzoic acid. Int. J. Mass Spectrom..

[53] Ng S.W. (2011). A triclinic modification of 3,4-dihydroxybenzoic acid monohydrate. Acta Cryst..

[54] Sarma B., Sanphui P., Nangia A. (2010). Polymorphism in isomeric dihydroxybenzoic acids. Cryst. Growth Des..

[55] Borbulevych O.Y., Jankun J., Selman S.H., Skrzypczak-Jankun E. (2004). Lipoxygenase interactions with natural flavonoid, quercetin, reveal a complex with protocatechuic acid in its X-ray structure at 2.1 Å resolution. Proteins: Struct. Funct. Bioinf..

[56] Zheng Y., Haworth I.S., Zuo Z., Chow M.S., Chow A.H. (2005). Physicochemical and structural characterization of quercetin-β-cyclodextrin complexes. J. Pharm. Sci..

[57] Bruker (2014). APEX2, SADABS and SHELXTL.

[58] Bruker (2008). SAINT and XPREP.

[59] Aree T., Jongrungruangchok S. (2016). Enhancement of antioxidant activity of green tea epicatechins in β-cyclodextrin cavity: Single-crystal X-ray analysis, DFT calculation and DPPH assay. Carbohydr. Polym..

[60] Schnupf U., Momany F.A. (2011). DFT energy optimization of a large carbohydrate: Cyclomaltohexaicosaose (CA-26). J. Phys. Chem. B.

[61] Allen F.H., Bruno I.J. (2010). Bond lengths in organic and metal-organic compounds revisited: X—H bond lengths from neutron diffraction data. Acta Cryst..

[62] Frisch M.J., Trucks G.W., Schlegel H.B., Scuseria G.E., Robb M.A., Cheeseman G., Scalmani V., Barone G.A., Petersson H., Nakatsuji (2009). GAUSSIAN09, Revision A.01.

[63] Salvatierra D., Jaime C., Virgili A., Sánchez-Ferrando F. (1996). Determination of the inclusion geometry for the β-cyclodextrin/benzoic acid complex by NMR and molecular modeling. J. Org. Chem..

[64] Aree T., Chaichit N. (2003). Crystal structure of β-cyclodextrin–benzoic acid inclusion complex. Carbohydr. Res..

[65] Aree T., Chaichit N., Engkakul C. (2008). Polymorphism in β-cyclodextrin–benzoic acid inclusion complex: A kinetically controlled crystal growth according to the Ostwald’s rule. Carbohydr. Res..

