# Supplementary material for: β-Cyclodextrin Inclusion Complexes with Catechol-Containing Antioxidants Protocatechuic Aldehyde and Protocatechuic Acid—An Atomistic Perspective on Structural and Thermodynamic Stabilities
Source: Molecules. 2021 Jun 11;26(12):3574. doi: 10.3390/molecules26123574 (PMC8230902; doi:10.3390/molecules26123574)
Supplement: Supplementary file 1 [file molecules-26-03574-s001.zip › molecules-1248199-supplementary.pdf]

Supplementary Materials

β-Cyclodextrin Inclusion Complexes with Catechol-Containing Antioxidants Protocatechuic Aldehyde and Protocatechuic Acid—An Atomistic Perspective on Structural and Thermodynamic Stabilities

Thammarat Aree

Department of Chemistry, Faculty of Science, Chulalongkorn University, Bangkok 10330, Thailand; Tel. +66-2-2187584; Fax +66-2-2187598; E-mail: athammar@chula.ac.th

Supplementary Materials Available:

I. Crystallographic Data

Table S1. X-ray Single Crystal Data Collection and Refinement Statistics of **1** and **2**.

Table S2. Comparison of β-CD Geometrical Parameters in **1**, **2**, β-CD·12H_2O and β-CD·(–)-epicatechin·4.2H_2O.

Table S3. O–H···O Hydrogen Bonds in β-CD·PCAL·6H_2O (**1**).

Table S4. O–H···O Hydrogen Bonds in β-CD·PCAC·6H_2O (**2**).

II. Computational Data

Fig. S1. Inclusion Complexes of (a) β-CD−PCAL and (b) β-CD−PCAC, Derived from DFT Complete-Geometry Optimization in the Gas Phase.

Table S5. O–H···O Hydrogen Bonds in β-CD–PCAL and β-CD–PCAC Inclusion Complexes from DFT Full-Geometry Optimization.

Table S6. Stabilization and Interaction Energies of β-CD–PCAL and β-CD–PCAC Complexes Compared to Other β-CD–3,4-dihydroxybenzene Complexes from DFT Full-Geometry Optimization.

III. References

I. Crystallographic Data

Table S1. X-ray Single Crystal Data Collection and Refinement Statistics of **1** and **2**.

	**1**<br>β-CD–Protocatechuic aldehyde	**2**<br>β-CD–Protocatechuic acid
Abbreviated formula	β-CD·PCAL·6H_2O	β-CD·PCAC·6H_2O
Chemical formula	$(C_6H_{10}O_5)_7 \cdot C_7H_6O_3 \cdot 6H_2O$	$(C_6H_{10}O_5)_7 \cdot C_7H_6O_4 \cdot 6H_2O$
Formula weight	1381.19	1397.19
Crystal habit, color	Thin plate, colorless	Thin plate, colorless
Crystal size [mm]	0.02 × 0.24 × 0.32	0.02 × 0.14 × 0.40
Crystal system, space group	Monoclinic, $P2_1$ (No. 4)	Monoclinic, $P2_1$ (No. 4)
Unit cell constants		
a [Å]	15.4213(11)	12.5165(7)
b [Å]	10.0440(7)	19.0287(12)
c [Å]	21.2795(15)	14.0626(8)
α [°]	90	90
β [°]	110.281(2)	109.656(2)
γ [°]	90	90
V [Å^3]	3091.7(4)	3154.2(3)
Z	2	2
D_c [g cm^{-3}]	1.484	1.471
μ [mm^{-1}]	0.133	0.132
F(000)	1468	1484
Diffractometer	APEXII Kappa CCD (Bruker)	APEXII Kappa CCD (Bruker)
Wavelength [Å]	MoK_α, 0.71073	MoK_α, 0.71073
T [K]	296(2)	296(2)
Data collection	ω–ϕ scan, 1.2° step, 8 s expose	ω–ϕ scan, 1.2° step, 10 s expose
Frames collected	600	680
θ range [°]	1.41–30.67	1.54–30.61
Resolution [Å]	0.70	0.70
Completeness [%], R_{int}	99.4, 0.0990	99.8, 0.0809
Reflns collected / unique / > 2$\sigma(I)$	60310 / 18989 / 7817	55384 / 19288 / 9845
Data / restraints / parameters	18989 / 18 / 860	19288 / 11 / 871
R_1, wR_2 [$I > 2\sigma(I)$][a]	0.0620, 0.1100	0.0602, 0.0879
R_1, wR_2 [all data][b], GoF	0.1927, 0.1527, 0.948	0.1509, 0.1134, 0.974
$\Delta\rho_{min}$, $\Delta\rho_{max}$ (e Å^{-3})	–0.28, 0.38	–0.34, 0.24
CCDC number	2075723	2075722

[a] $R = \sum||F_o| - |F_c||/\sum|F_o|$.
[b] $wR = \sum\{w(F_o^2 - F_c^2)^2/\sum w(F_o^2)^2\}^{1/2}$.

Table S2. Comparison of β-CD Geometrical Parameters in **1**, **2**, β-CD·12H_2O and β-CD·(–)-epicatechin·4.2H_2O.[a]

Residue	Puckering Q [Å] [b], θ [º] [c]				Tilt angle [º] [d]				O4 deviation [Å] [e]				O4(n)···O4(n – 1), O4(n)···centroid [Å]			
n	**1**	**2**	**β-CD ·12W**	**β-CD ·EC**	**1**	**2**	**β-CD ·12W**	**β-CD ·EC**	**1**	**2**	**β-CD ·12W**	**β-CD ·EC**	**1**	**2**	**β-CD ·12W**	**β-CD ·EC**
1	0.574(6)	0.573(4)	0.571	0.557(2)									4.479(5)	4.425(4)	4.247	4.498(2)
	3.1(6)	5.4(4)	3.9	4.9(3)	22.9(3)	23.1(1)	20.3	33.7(1)	–0.150(3)	0.125(2)	–0.115	–0.199(1)	5.192	4.531	5.184	4.827
2	0.543(6)	0.562(4)	0.567	0.578(2)									4.182(5)	4.415(4)	4.338	4.322(2)
	6.0(6)	0.0(4)	3.7	3.2(2)	14.2(3)	11.3(1)	6.4	1.9(1)	0.290(3)	0.313(2)	–0.196	–0.073(1)	5.319	5.300	4.913	5.435
3	0.585(6)	0.572(4)	0.570	0.565(2)									4.363(5)	4.117(4)	4.489	4.209(2)
	1.7(6)	7.5(4)	7.6	4.9(2)	3.3(2)	11.8(2)	15.0	14.6(1)	0.050(3)	–0.392(2)	0.192	0.087(1)	4.638	5.213	4.981	5.075
4	0.592(6)	0.558(5)	0.583	0.582(2)									4.570(5)	4.425(4)	4.392	4.626(2)
	7.7(6)	3.7(5)	3.0	8.8(2)	18.5(3)	30.3(2)	26.2	30.6(1)	–0.378(3)	–0.022(2)	0.091	0.124(1)	4.896	4.608	5.153	4.632
5	0.543(6)	0.573(4)	0.559	0.561(2)									4.154(5)	4.562(4)	4.286	4.403(2)
	9.7(6)	9.5(4)	3.9	6.7(2)	24.5(2)	8.9(2)	10.8	19.7(1)	0.188(3)	0.312(2)	–0.195	–0.180(1)	5.382	5.051	5.122	5.171
6	0.550(6)	0.563(4)	0.596	0.545(2)									4.318(5)	4.309(4)	4.443	4.263(2)
	3.5(6)	1.9(4)	1.4	5.6(2)	3.4(3)	11.6(2)	7.9	4.6(1)	0.223(3)	–0.095(2)	–0.053	–0.084(1)	4.960	5.356	4.856	5.397
7	0.569(6)	0.573(4)	0.579	0.574(2)									4.523(5)	4.289(4)	4.452	4.278(2)
	8.1(6)	2.6(4)	2.0	3.7(2)	20.5(3)	11.4(3)	10.7	6.9(1)	–0.224(3)	–0.240(2)	0.276	0.325(1)	4.736	4.963	5.054	4.581
													0.416 [f]	*0.445*	*0.242*	*0.417*
													0.744 [f]	*0.825*	*0.328*	*0.854*
													0.874 [f]	*0.876*	*0.870*	*0.876*

[a] β-CD·12H_2O [1] and β-CD·(–)-epicatechin·4.2H_2O [2].

[b,c] An ideal cyclohexane chair (for R(C–C) = 1.54 Å) has puckering amplitude Q = 0.63 Å and angle describing the polar position θ = 0º [3].

[d] Interplanar angle of the plane through C1(n), C4(n), O4(n) and O4(n – 1) against the O4 plane.

[e] Deviation of glycosidic O4 atoms from the least-squares plane through the seven O4 atoms.

[f] Ranges of the O4(n)···O4(n – 1), O4(n)···centroid distances and the average of their ratios are in *italics*; for an ideal heptagon, the ratio is 0.868.

[g] Endocyclic torsion angles ϕ and ψ at glycosidic O4, defined as O5(n + 1)–C1(n + 1)–O4(n)–C4(n) and C1(n + 1)–O4(n)–C4(n)–C5(n), respectively.

[h] Averages of ϕ and ψ are in *italics*; for the CD roundness, the sum of averages should be nearly zero [4].

[i] Exocyclic torsion angles χ and ω are defined as C4–C5–C6–O6 and O5–C5–C6–O6, respectively.

Table S2. Continued.

Residue	O3(*n*)···O2(*n* + 1) distance [Å]				Torsion angles ϕ [g], ψ [g] [°]				Torsion angles χ [i], ω [i] [°]			
n	**1**	**2**	**β-CD ·12W**	**β-CD ·EC**	**1**	**2**	**β-CD ·12W**	**β-CD ·EC**	**1**	**2**	**β-CD ·12W**	**β-CD ·EC**
1					108.1(5)	116.2(4)	120.0	129.1(2)	47.6(6)	177.8(3)	–175.4	55.7(3)
	2.811(6)	2.731(4)	2.855	3.346(3)	–113.4(5)	–103.1(4)	–109.8	–97.4(2)	–72.7(6)	60.4(4)	64.7	–64.5(2)
2					106.1(4)	103.9(4)	103.0	90.1(2)	61.9(6)	57.0(4)	52.1	60.7(2)
	2.939(5)	2.893(4)	2.862	2.828(3)	–117.6(5)	–141.1(3)	–125.7	–131.9(2)	–59.4(6)	–62.7(4)	–62.9	–60.9(2)
3					102.5(5)	115.4(4)	119.3	117.1(2)	58.3(6)	–168.0(3)	–169.4	–167.8(2)
	2.896(5)	2.841(5)	2.957	3.246(3)	–112.2(5)	–105.8(4)	–95.9	–105.9(2)	–63.5(6)	70.5(4)	70.5	69.8(2)
4					115.1(5)	122.6(4)	110.5	105.9(2)	45.9(7)	55.7(5)	–173.9	54.2(3)
	2.810(6)	2.982(5)	2.875	2.833(3)	–102.8(5)	–102.8(4)	–106.6	–96.5(2)	–73.7(6)	–64.2(5)	71.0	–64.7(3)
5					117.4(4)	99.2(4)	102.5	102.7(2)	–171.6(5)	56.9(5)	58.7	53.5(2)
	3.133(6)	3.056(4)	2.902	2.924(2)	–111.4(5)	–125.6(4)	–121.1	–118.4(2)	67.1(6)	–63.7(4)	–60.8	–66.8(2)
6					97.9(5)	101.4(4)	107.7	100.7(2)	53.4(6)	61.6(5)	57.0	58.7(3)
	2.992(6)	2.729(4)	2.783	2.765(2)	–120.4(5)	–109.9(4)	–109.4	–121.5(2)	–66.7(5)	–60.3(4)	–61.0	–62.7(2)
7					114.0(5)	112.5(4)	110.7	111.6(2)	54.9(7)	–177.2(3)	50.7	48.9(2)
	2.803(6)	2.802(4)	2.770	2.762(2)	–95.4(5)	–116.1(4)	–114.1	–118.1(2)	–65.1(6)	62.0(4)	–71.0	–72.5(2)
					108.7 [h]	*110.2*	*110.5*	*108.2*				
					–110.5 [h]	*–114.9*	*–111.8*	*–112.8*				
					–1.7 [h]	*–4.7*	*–1.3*	*–4.6*				

[a] β-CD·12H_2O [1] and β-CD·(–)-epicatechin·4.2H_2O [2].

[b,c] An ideal cyclohexane chair (for *R*(C–C) = 1.54 Å) has puckering amplitude Q = 0.63 Å and angle describing the polar position θ = 0° [3].

[d] Interplanar angle of the plane through C1(*n*), C4(*n*), O4(*n*) and O4(*n* – 1) against the O4 plane.

[e] Deviation of glycosidic O4 atoms from the least-squares plane through the seven O4 atoms.

[f] Ranges of the O4(*n*)···O4(*n* – 1), O4(*n*)···centroid distances and the average of their ratios are in *italics*; for an ideal heptagon, the ratio is 0.868.

[g] Endocyclic torsion angles ϕ and ψ at glycosidic O4, defined as O5(*n* + 1)–C1(*n* + 1)–O4(*n*)–C4(*n*) and C1(*n* + 1)–O4(*n*)–C4(*n*)–C5(*n*), respectively.

[h] Averages of ϕ and ψ are in *italics*; for the CD roundness, the sum of averages should be nearly zero [4].

[i] Exocyclic torsion angles χ and ω are defined as C4–C5–C6–O6 and O5–C5–C6–O6, respectively.

Table S3. O–H···O Hydrogen Bonds in β-CD·PCAL·6H_2O (**1**) [Å, °].

Interaction	O–H	O···O	∠(OHO)	Interaction	O–H	O···O	∠(OHO)
β-*CD*–β-*CD* [a]							
O21–H···O37	0.82	2.803(6)	163.3	O4W–H1···O63	0.95	2.687(6)	175.9
O22–H···O31	0.82	2.811(6)	159.1	O5W–H1···O34 [x]	0.95	2.929(6)	172.3
O32–H···O23	0.82	2.939(5)	177.5	O6W–H2···O64	0.96	2.815(7)	135.7
O24–H···O33	0.82	2.896(5)	165.4	O64–H···O5W	0.82	2.725(7)	171.2
O34–H···O25	0.82	2.810(6)	167.3	O25–H···O4W [vi]	0.82	2.709(5)	173.0
O35–H···O26	0.82	3.133(6)	170.8	O1W–H2···O25 [iii]	0.94	2.891(5)	136.1
O27–H···O36	0.82	2.993(6)	162.8	O65–H···O1W	0.82	2.741(6)	115.9
O31–H···O51 [i c]	0.82	3.208(6)	129.7	O6W–H1···O65	0.93	2.775(6)	155.1
O61–H···O21 [ii]	0.82	2.822(6)	141.4	O26–H···O3W [viii]	0.82	2.781(6)	146.0
O33–H···O61 [v]	0.82	2.862(6)	150.5	O66–H···O3W [vii]	0.82	2.848(6)	138.3
O63–H···O56 [iv]	0.82	3.098(6)	128.7	O2W–H1···O27 [i]	0.94	2.962(6)	124.9
O63–H···O66 [iv]	0.82	2.766(6)	127.0	*PCAL*–β-*CD*/H_2O			
O36–H···O22 [i]	0.82	2.727(6)	151.5	O2L–H···O1L	0.82	2.700(6)	114.1
O37–H···O23 [ii]	0.82	3.053(6)	129.6	O1L–H···O1W	0.82	2.658(6)	177.7
O67–H···O26 [iii]	0.82	2.766(6)	163.4	O2L–H···O34 [iii]	0.82	2.874(6)	166.0
β-*CD*–H_2O [b]				O31–H···O3L [ii]	0.82	2.842(6)	134.4
O2W–H2···O32 [ix]	0.96	2.826(6)	141.6	H_2O–H_2O			
O3W–H2···O62	0.95	2.847(7)	144.2	O3W–H1···O6W [x]	0.95	2.829(7)	121.4
O62–H···O2W [iii]	0.82	3.102(7)	144.2	O4W–H2···O6W [vi]	0.95	2.919(6)	168.3
O23–H···O2W	0.82	2.710(6)	176.2	O5W–H2···O4W	0.95	3.092(7)	148.8

[a] β-CD without doubly disordered O6–H groups.
[b] Six fully occupied water molecules.
[c] Equivalent positions: (i) –*x* + 1, *y* + 0.5, –*z* + 2; (ii) –*x* + 1, *y* – 0.5, –*z* + 2; (iii) *x*, *y* – 1, *z*; (iv) *x* + 1, *y*, *z*; (v) *x*, *y* + 1, *z*; (vi) –*x* + 1, *y* + 0.5, –*z* + 1; (vii) *x* – 1, *y*, *z*; (viii) *x* – 1, *y* + 1, *z*; (ix) –*x* + 2, *y* + 0.5, –*z* + 2; (x) –*x* + 1, *y* – 0.5, –*z* + 1.

Table S4. O–H···O Hydrogen Bonds in β-CD·PCAC·6H_2O (**2**) [Å, °].

Interaction	O–H	O···O	∠(OHO)	Interaction	O–H	O···O	∠(OHO)
β-*CD*–β-*CD* [a]				O2W–H1···O63	0.96	2.911(5)	156.3
O21–H···O37	0.82	2.802(4)	157.9	O3W–H2···O63 [ix]	0.96	2.845(5)	154.5
O31–H···O22	0.82	2.731(4)	173.0	O4W–H1···O64 [ix]	0.97	2.785(5)	168.5
O23–H···O32	0.82	2.893(4)	164.1	O4W–H2···O55 [ix]	0.95	2.965(5)	115.6
O24–H···O33	0.82	2.841(5)	165.6	O5W–H1···O65	0.96	2.757(5)	171.1
O34–H···O25	0.82	2.982(5)	169.4	O26–H···O5W [vii]	0.82	2.975(5)	157.3
O35–H···O26	0.82	3.056(4)	175.8	O6W–H1···O36	0.96	2.768(5)	173.6
O36–H···O27	0.82	2.729(4)	158.4	O3W–H1···O66	0.96	2.754(5)	174.5
O61–H···O23 [i c]	0.82	2.934(4)	165.2	O27–H···O1W [ix]	0.82	2.803(5)	164.2
O62–H···O21 [ii]	0.82	2.817(4)	153.9	O1W–H1···O67	0.96	2.864(4)	154.6
O33–H···O67 [iii]	0.82	2.765(4)	174.7	*PCAC*–β-*CD*/H_2O			
O63–H···O31 [ii]	0.82	2.790(4)	158.9	O1D–H···O2D	0.82	2.703(5)	108.7
O25–H···O52 [v]	0.82	2.855(4)	161.0	O1D–H···O1W	0.82	2.705(5)	168.9
O65–H···O21 [vi]	0.82	2.767(4)	169.1	O2D–H···O2W	0.82	2.670(6)	167.3
O66–H···O52 [viii]	0.82	3.073(4)	120.6	O64–H···O3D [iv]	0.82	2.937(6)	167.4
O66–H···O62 [viii]	0.82	2.849(5)	167.0	O67–H···O3D [i]	0.82	2.767(5)	154.3
O37–H···O24 [x]	0.82	2.712(4)	149.1	O4D–H···O3W [iii]	0.82	2.631(5)	171.5
β-*CD*–H_2O [b]				H_2O–H_2O			
O1W–H2···O61	0.96	3.044(5)	169.6	O2W–H2···O6W [vi]	0.96	2.869(5)	162.2
O6W–H2···O61 [ix]	0.96	2.857(5)	174.3	O4W–H2···O5W [ix]	0.95	2.829(6)	149.5
O22–H···O4W [iii]	0.82	2.841(5)	166.8	O5W–H2···O6W [xi]	0.96	2.936(5)	166.7
O32–H···O4W [iii]	0.82	2.678(5)	167.9				

[a] β-CD without doubly disordered O6–H groups.
[b] Six fully occupied water molecules.
[c] Equivalent positions: (i) *x* – 1, *y*, *z*; (ii) –*x* + 2, *y* – 0.5, –*z* + 2; (iii) *x* + 1, *y*, *z*; (iv) –*x* + 2, *y* – 0.5, –*z* + 1; (v) *x*, *y*, *z* – 1; (vi) –*x* + 1, *y* – 0.5, –*z* + 1; (vii) –*x* + 1, *y* + 0.5, –*z*; (viii) *x* – 1, *y*, *z* – 1; (ix) –*x* + 1, *y* + 0.5, –*z* + 1; (x) –*x* + 2, *y* + 0.5, –*z* + 1; (xi) –*x* + 1, *y* – 0.5, –*z*.

II. Computational Data

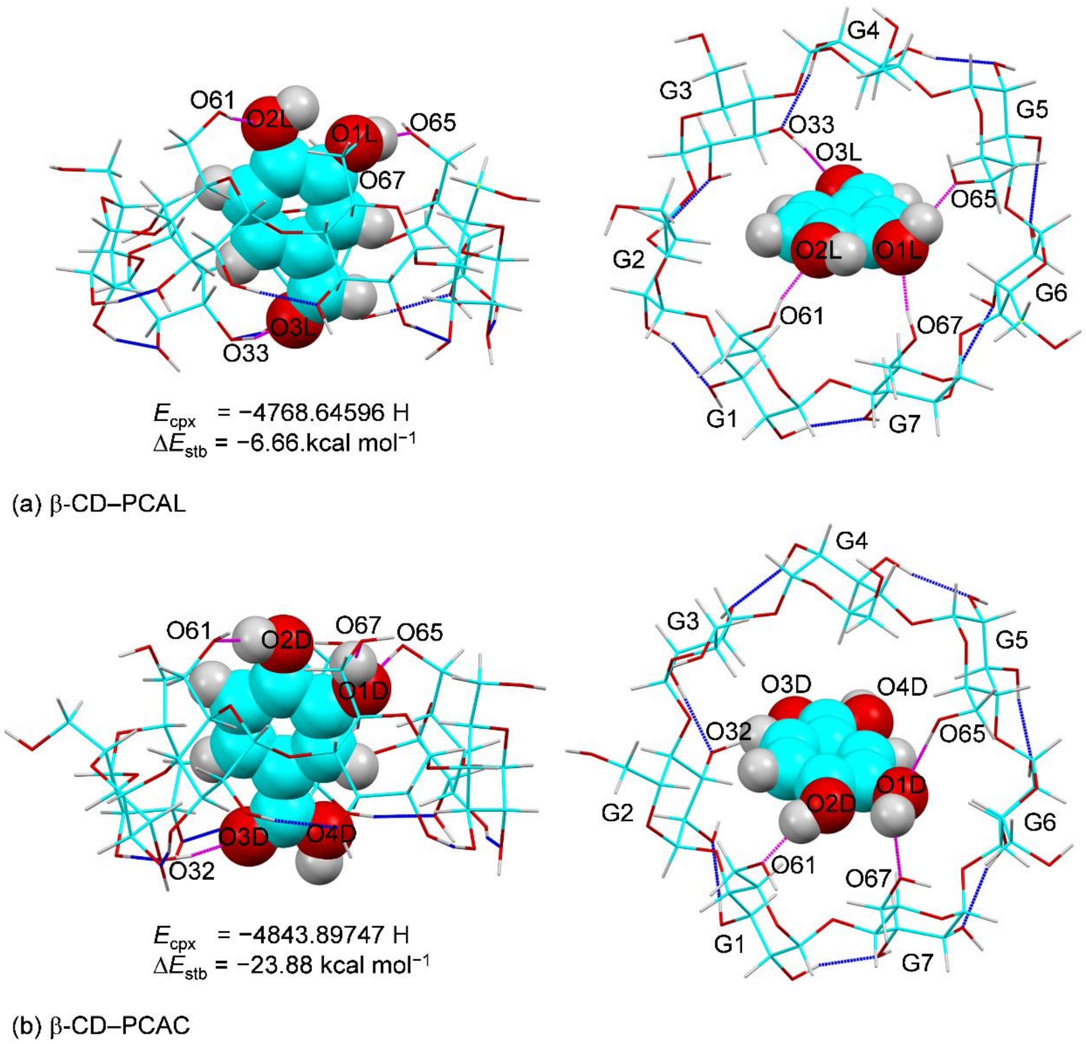

Fig. S1. Inclusion complexes of (a) β-CD−PCAL and (b) β-CD−PCAC, derived from DFT complete-geometry optimization in the gas phase; side view (left) and top view (right). For better comparison, the energy of complex (E_{cpx}) and stabilization energy (ΔE_{stb}) are given; see also Tables S5. The intramolecular O3(n)···O2(n + 1) H-bonds of β-CD and host–guest O–H···O H-bonds are indicated by blue and magenta connecting lines, respectively.

Table S5. O–H···O Hydrogen Bonds in β-CD–PCAL and β-CD–PCAC Inclusion Complexes from DFT Full-Geometry Optimization [Å, °]. [a]

Interaction	O–H	O···O	∠(OHO)	Interaction	O–H	O···O	∠(OHO)
β-CD·PCAL [b]		β–CD	conformation				
β-*CD*–β-*CD*		Distorted	round	*PCAL*–β-*CD*			
O21–H···O37	0.98	2.82	164.9	O2L–H···O1L	0.98	2.69	114.4
O22–H···O31	0.98	2.84	160.9	O61–H···O2L	0.97	2.95	164.7
O32–H···O23	0.98	2.86	158.3	O33–H···O3L	0.98	2.93	164.7
O24–H···O33	0.98	2.94	168.3	O67–H···O1L	0.97	3.02	169.3
O34–H···O25	0.98	2.92	166.0	O1L–H···O65	0.99	2.74	177.8
O35–H···O26	0.98	2.90	162.6				
O27–H···O36	0.98	3.26	167.0				
β-CD·PCAC [b]							
β-*CD*–β-*CD*		Distorted	round	*PCAC*–β-*CD*			
O21–H···O37	0.98	2.87	159.6	O1D–H···O2D	0.99	2.69	108.7
O31–H···O22	0.98	2.87	154.4	O65–H···O1D	0.97	3.02	177.5
O23–H···O32	0.98	2.92	177.8	O1D–H···O67	0.99	3.01	145.1
O24–H···O33	0.98	2.97	158.6	O2D–H···O61	0.99	2.78	161.9
O34–H···O25	0.98	2.93	167.1	O32–H···O3D	0.98	2.90	169.4
O35–H···O26	0.98	2.94	165.5				
O36–H···O27	0.98	3.03	162.3				

[a] DFT energy minimization in vacuum at B3LYP/6–31+G*/4–31G level, see also Fig. S1 and Table S6.
[b] X-ray-derived structures were used as starting models.

Table S6. Stabilization and Interaction Energies of β-CD–PCAL and β-CD–PCAC Complexes Compared to Other β-CD–3,4-dihydroxybenzene Complexes from DFT Full-Geometry Optimization. [a]

	β-CD–PCAL	β-CD–PCAC	β-CD–HTY [b]	β-CD–OLE [b]	β-CD–CFA [c]	β-CD–CGA [c]
E_{cpx} [d]	−4768.64596	−4843.89747	−4809.11016	−6221.48698	−5569.72645	−4921.19194
$E_{\beta\text{-CD\_opt}}$ [e]	−4272.97625	−4272.95438	−4272.95853	−4272.95892	−4272.96989	−4272.96123
E_{G_opt}	−495.65910	−570.90503	−536.12216	−1948.47613	−1296.72905	−648.20786
$E_{\beta\text{-CD\_sp}}$	−4272.96945	−4272.94828	−4272.95412	−4272.94227	−4272.96725	−4272.95827
E_{G_sp}	−495.65651	−570.90054	−536.11813	−1948.46783	−1296.72722	−648.20576
ΔE_{stb} [Hartree] [f]	−0.01061	−0.03806	−0.02948	−0.05193	−0.02751	−0.02285
ΔE_{stb} [kcal mol^{-1}]	−6.66	−23.88	−18.50	−32.58	−17.26	−14.34
$\Delta\Delta E_{stb}$ [kcal mol^{-1}] [h]	25.92	8.7	14.08	0	15.32	18.24
ΔE_{int} [Hartree] [g]	−0.02000	−0.04865	−0.03792	−0.07688	−0.02792	−0.03198
ΔE_{int} [kcal mol^{-1}]	−12.55	−30.53	−23.79	−48.24	−17.52	−20.07
$\Delta\Delta E_{int}$ [kcal mol^{-1}] [i]	35.69	17.71	24.45	0	30.72	28.17
No. of host-guest OH···O hydrogen bonds	4	4	3	6	1	2

[a] DFT/B3LYP calculations in the gas phase with mixed basis sets 4–31G for C atoms and 6–31+G* for O atoms were carried out using program GAUSSIAN09 [5]. X-ray-derived structures were used as starting models, see also Fig. S1 and Table S5.
[b] Ref. [6].
[c] Ref. [7].
[d] Original unit of E is Hartree [1 H = 627.5 kcal mol^{-1}].
[e] $E_{\beta\text{-CD\_opt}}$ in vacuum of the uncomplexed β-CD·12H_2O [1] is −4272.96662 H.
[f,g] Stabilization energy, $\Delta E_{stb} = E_{cpx} - (E_{\beta\text{-CD\_opt}} + E_{G\_opt})$
Interaction energy, $\Delta E_{int} = E_{cpx} - (E_{\beta\text{-CD\_sp}} + E_{G\_sp})$, where E_{cpx}, $E_{\beta\text{-CD\_opt}}$ and E_{G_opt} are the energies from full optimization of complex, host β-CD and guest PCAL/PCAC, respectively; $E_{\beta\text{-CD\_sp}}$ and E_{G_sp} are the corresponding single-point energies in the complexed states.
[h,i] Relative stabilization energy and relative interaction energy ($\Delta\Delta E_{stb}$ and $\Delta\Delta E_{int}$) calculated using the most stable complex β-CD–OLE [6] as a reference.

Note: No basis set superposition error (BSSE) correction is applied to the DFT-derived energies of the β-CD–catechol antioxidant complexes because the estimated energy differences ($\Delta\Delta E_{stb}$ and $\Delta\Delta E_{int}$) are sufficient to interpret the relative thermodynamic stabilities in relation to host–guest interactions and antioxidant properties.

III. References

1. Lindner, K.; Saenger, W. Crystal and molecular structure of cyclohepta-amylose dodecahydrate. *Carbohydr. Res.* **1982**, *99*, 103–115. https://doi.org/10.1016/S0008-6215(00)81901-1.
2. Aree, T.; Jongrungruangchok, S. Crystallographic evidence for β-cyclodextrin inclusion complexation facilitating the improvement of antioxidant activity of tea (+)-catechin and (−)-epicatechin. *Carbohydr. Polym.* **2016**, *140*, 362–373. https://doi.org/10.1016/j.carbpol.2015.12.066.
3. Cremer, D.T.; Pople, J.A. General definition of ring puckering coordinates. *J. Am. Chem. Soc.* **1975**, *97*, 1354–1358. https://doi.org/10.1021/ja00839a011.
4. French, A.D.; Johnson, G.P. Linkage and pyranosyl ring twisting in cyclodextrins. *Carbohydr. Res.* **2007**, *342*, 1223–1237. https://doi.org/10.1016/j.carres.2007.02.033.
5. Frisch, M.J.E.A.; Trucks, G.W.; Schlegel, H.B.; Scuseria, G.E.; Robb, M.A.; Cheeseman, J.R.; ... Nakatsuji, H. GAUSSIAN09, Revision A.01. Gaussian. Inc., Wallingford, CT. 2009.
6. Aree, T.; Jongrungruangchok, S. Structure–antioxidant activity relationship of β-cyclodextrin inclusion complexes with olive tyrosol, hydroxytyrosol and oleuropein: Deep insights from X-ray analysis, DFT calculation and DPPH assay. *Carbohydr. Polym.* **2018**, *199*, 661–669. https://doi.org/10.1016/j.carbpol.2018.07.019.
7. Aree, T. Understanding structures and thermodynamics of β-cyclodextrin encapsulation of chlorogenic, caffeic and quinic acids: Implications for enriching antioxidant capacity and masking bitterness in coffee. *Food Chem.* **2019**, *293*, 550–560. https://doi.org/10.1016/j.foodchem.2019.04.084.
